# Spatial Distribution and Interspecific Associations of Tree Species in a Tropical Seasonal Rain Forest of China

**DOI:** 10.1371/journal.pone.0046074

**Published:** 2012-09-28

**Authors:** Guoyu Lan, Stephan Getzin, Thorsten Wiegand, Yuehua Hu, Guishui Xie, Hua Zhu, Min Cao

**Affiliations:** 1 Key Laboratory of Tropical Forest Ecology, Xishuangbanna Tropical Botanical Garden, Chinese Academy of Sciences, Kunming, P. R. China; 2 Rubber Research Institute, The Chinese Academy of Tropical Agricultural Sciences, Danzhou City, Hainan Province, P. R. China; 3 Department of Ecological Modelling, UFZ Helmholtz Centre for Environmental Research, Leipzig, Germany; Utah State University, United States of America

## Abstract

Studying the spatial pattern and interspecific associations of plant species may provide valuable insights into processes and mechanisms that maintain species coexistence. Point pattern analysis was used to analyze the spatial distribution patterns of twenty dominant tree species, their interspecific spatial associations and changes across life stages in a 20-ha permanent plot of seasonal tropical rainforest in Xishuangbanna, China, to test mechanisms maintaining species coexistence. Torus-translation tests were used to quantify positive or negative associations of the species to topographic habitats. The results showed: (1) fourteen of the twenty tree species were negatively (or positively) associated with one or two of the topographic variables, which evidences that the niche contributes to the spatial pattern of these species. (2) Most saplings of the study species showed a significantly clumped distribution at small scales (0–10 m) which was lost at larger scales (10–30 m). (3) The degree of spatial clumping deceases from saplings, to poles, to adults indicates that density-dependent mortality of the offspring is ubiquitous in species. (4) It is notable that a high number of positive small-scale interactions were found among the twenty species. For saplings, 42.6% of all combinations of species pairs showed positive associations at neighborhood scales up to five meters, but only 38.4% were negative. For poles and adults, positive associations at these distances still made up 45.5% and 29.5%, respectively. In conclusion, there is considerable evidence for the presence of positive interactions among the tree species, which suggests that species herd protection may occur in our plot. In addition, niche assembly and limited dispersal (likely) contribute to the spatial patterns of tree species in the tropical seasonal rain forest in Xishuangbanna, China.

## Introduction

Numerous mechanisms have been proposed to explain coexistence of tree species in tropical forests on local scales [1]–[2]. One of the most prominent hypotheses is the Janzen–Connell hypothesis that states that distance- or density-dependent mortality due to predation or host-specific pests should promote less aggregated and more mingled spatial distributions of species [3]–[4]. Condit et al. [5] suggested that the role of density-dependence may only be important among those species with the highest population densities. Later research showed that density-dependence was very common in some tropical forests 6–8. An extension of the Janzen–Connell hypothesis, the species herd protection hypothesis, suggests that heterospecifc neighbors can promote coexistence by thwarting the transmission of biotic plant pests [7], [9]. According to the species-herd protection hypothesis, heterospecific crowding may be of general benefit for the survival of recently established seedlings because fewer encounters between a host and its species-specific pests and pathogens would occur [9]–[10].

The Janzen–Connell and the species herd protection hypotheses make specific predictions on the spatial placement of individuals of different species. First, the spatial pattern of individual species should become with progressing life stage less aggregated because high density clumps of a given species are more prone to predation or host-specific pests. A similar pattern is expected due to self thinning [11]. However, the mechanism of the herd protection hypothesis may additionally create or maintain positive spatial associations among species despite the expected effect of interspecific competition. Thus, we expect for progressing life stages that the species patterns should become less aggregated and the occurrence of positive associations among species should increase (or be maintained). One possibility to explore if observed spatial placement of trees is compatible with this hypothesis is to use techniques of spatial point pattern analysis [12]–[14].

The dipterocarp tropical seasonal rain forest is one the most important vegetation types in southwest China and harbors biodiversity that is important both for global and national species richness in China. The 20-ha Xishuangbanna forest dynamics plot was established in 2007 in this vegetation type to test theories and hypotheses related to biodiversity maintenance. For example, of special interest is to find evidence (direct or indirect) of Janzen-Connell effects (or species herd protection), niche assembly or dispersal limitation [8]. Here we focus on the Janzen-Connell and species herd protection hypotheses and conduct a detailed spatial point pattern analysis of the dominant tree species in this forest. Studying the factors that structure the spatial pattern and relationship of the dominant species is of interest because they determine to a large extend the light climate in the forest and play a major role in structuring the lower layers of the forest. Because of their large size, tree species are also expected to exert positive or negative interactions to other tree species or plants at lower layers. For example, it is believed that the emergent tree species *Parashorea chinensis* wang hsie at Xishuangbanna could facilitate the survival of other plants by building up a humid microenvironment favorable in the dry season [15]. However, Kohyama [16] demonstrated for a warm-temperate rainforest that one-sided competition for light (i.e., light is intercepted mainly by larger trees and smaller trees have limited access to light) plays a key role in spatial pattern formation of trees. Whether these species promote or hamper each other to reach the light in the top layer remains an open question.

In this paper we used point pattern analysis to analyze the spatial species distributions, interspecific species associations and their change across life stages among the twenty dominant species of the Xishuangbanna forest dynamics plot. Our specific objectives were to explore if Janzen-Connell effects or species herd protection are potential mechanisms for structuring the species patterns in our study site. To this end, we formulate two non exclusive guiding hypotheses: (1) the univariate spatial pattern of the twenty dominant species should become more regular with increasing life stage and (2) the bivariate (interspecific) spatial patterns of the twenty dominant species should show positive association at small scales and all life stages. Finding evidence for hypothesis (1) would suggest operation of Janzen-Connell effects while evidence for (2) would agree with the species herd protection hypothesis and generally facilitation among different species.

## Methods

### Study site

**Figure 1:**
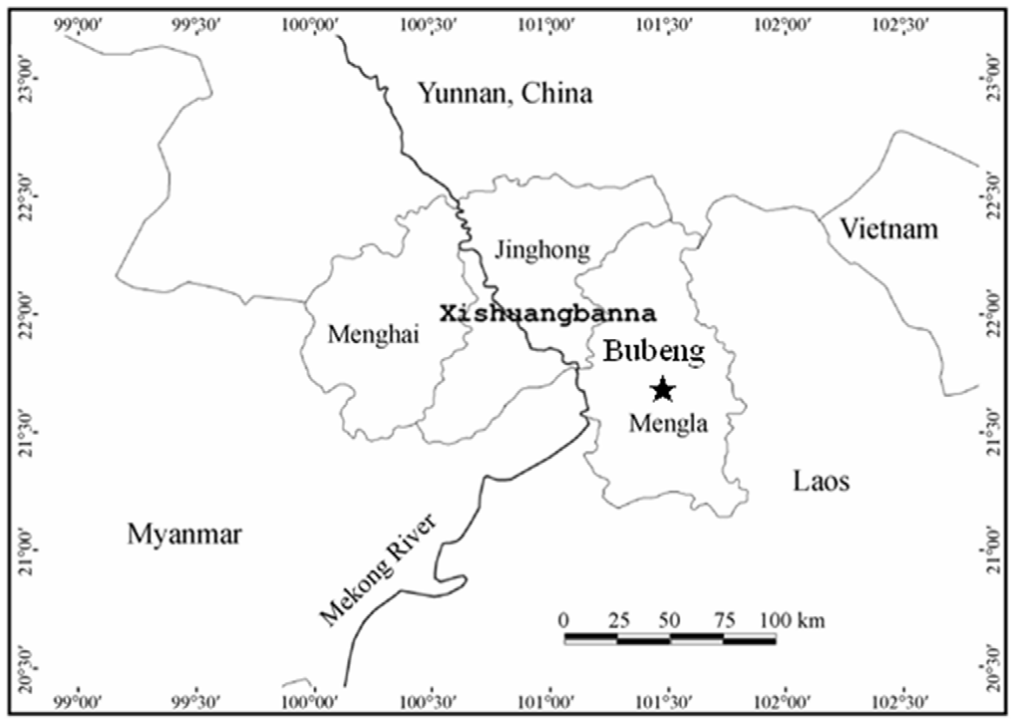
Location (marked with “★”) of the 20-ha plot in a tropical seasonal rainforest of China.

The study site was located in the Xishuangbanna National Nature Reserve in south-western China (101°34′E, 21°36′N) (Fig. 1). It borders Myanmar in the southwest and Laos in the southeast, and has mountainous topography, with mountain ridges running in a north–south direction, decreasing in elevation southward. The uplift of the Himalayas leads to the penetration of warm and moist tropical air mass from the Indian Ocean to Xishuangbanna in the summer, and forms a barrier preventing cold air mass from the north reaching the region in the winter, allowing for the existence of tropical seasonal rain forest in its altitudinal and latitudinal northern limits. The region is dominated by a typical monsoon climate with an alternation between a dry season and a rainy season. As recorded by a weather station 14 km away from the study site, the mean annual temperature is 21.0°C, and the mean annual precipitation is 1532 mm, of which approximately 80% occurs between May and October. The dry season is from November to April [17]. Under these climatic conditions, the tropical seasonal rain forest grows in the lowlands, valleys and hills that have a good water supply [15], [18]–[19].

### Data collection

A 20-ha permanent forest plot was established in the Xishuangbanna National Nature Reserve in 2007 following the field protocol of the Center for Tropical Forest Science [20]–[23]. The plot is rectangular in shape and measures 400 m (north-south) by 500 m (east-west). The elevation of the plot ranges from 709 m to 869 m above sea level; the highest elevation occurs in the north-west corner of the plot. Three perennial creeks run through the plot and join together in the south-eastern corner of the plot. The forest occurs mainly on laterite and lateritic red soils with pH values ranging from 4.5–5.5.

All trees (≥1 cm in diameter at breast height, DBH) were mapped and tagged with unique numbers. Tree diameters were measured 1.3 m from the ground. All stems were identified to species. The top tree layer with very uneven crown canopy reaches 30–60 m high and has a coverage of about 30%. As an emergent tree, the single dominant species *Parashorea chinensis* is the tallest tree, with crown branches near the top and its semi-orbicular crown soars high. Other top tree species such as *Sloanea tomentosa* (Benth.) Rehd. et Wils, *Pometia tomentosa* (Bl.) Teysm. et Binn, *Semecarpus reticulata* Lecte, *Barringtonia pendula* (Griff.) Kurz usually occupy a space of 30–45 m high above the continuous crown canopy of the second tree layer and under the crown of *Parashorea chinensis*. The second tree layer reaches up to 18–30 m high. *Garcinia cowa* predominates in this layer and other representative species are *Ficus langkokensis* Drake, *Knema furfuracea* (Hook.f. & Thomson) Warb., *Cinnamomum bejolghota* (Buch.-Ham.) Sweet, *Castanopsis echidnocarpa* Hook.f. & Thoms. ex Miq., The third tree layer is 6–20 m high and can be roughly divided into two sub-layers. The upper sub-layer occupies the 10–20 m high space and has as common species *Baccaurea ramiflora* Lour., *Dichapetalum genonioides* (Roxb.) Engl., *Castanopsis hystrix* Miq., *Castanopsis indica* (Roxb.) Miq., The lower sub-layer is 6–10 m high. The species *Pittosporopsis kerrii* Craib predominates and other common species are *Phoebe lanceolata* (Nees) Nees, *Mezzettiopsis creaghii* Ridl., *Leea compactiflora* Kurz, *Saprosma ternata*, , *Nephelium chryseum* Bl. [18], [24].

Relative density (FD), relative dominance (RA, using basal area) and relative frequency (RF) were calculated for each species in order to estimate the importance value (IV) [24]. We selected the top twenty tree species with the greatest importance values for our analysis. And these species comprised more than 60% of the total individuals (95,498) of trees ≥1.0 cm DBH (see Table 1).

**Table 1:** Species properties.

Rank	Species	Code	Family	Life type	Fruit type	Dispersal model	No. of individuals	Percentage (%)	No. of sapling	No. of poles	No. of adults	Habitat association
1	*Parashorea chinesis*	PARACH	Dipterocarpaceae	Emergent	Samara	Wind, Gravity	7919	8.3	6492	1276	151	High plateau−;Gap−
2	*Sloanea tomentosa*	SLOATO	Elaeocarpaceae	Upper canopy	Capsule	Ballistic	502	0.5	222	196	84	N
3	*Pometia tomentosa*	POMETO	Sapindaceae	Upper canopy	Drupe	Animal	480	0.5	274	147	59	Low-slope+; High-plateau−
4	*Semecarpus reticulata*	SEMERE	Anacardiaceae	Upper canopy	Drupe	Animal	619	0.6	354	234	31	High-plateau−
5	*Barringtonia pendula*	BARRPE	Lecythidaceae	Upper canopy	Drupe	Gravity	573	0.6	314	234	25	N
6	*Garcinia cowa*	GARCCO	Guttiferae	Lower canopy	Drupe	Animal, Gravity	4333	4.5	2795	1448	90	High-plateau+; Low-slope−
7	*Knema furfuracea*	KNEMFU	Myristicaceae	Lower canopy	Drupe	Animal	3160	3.3	2543	578	39	Gap+
8	*Ficus langkokensis*	FICULA	Moraceae	Lower canopy	Sycarp	Animal	1337	1.4	761	537	39	N
9	*Cinnamomum bejolghota*	CINNBE	Lauraceae	Lower canopy	Berry	Animal	1337	1.4	938	376	23	N
10	*Castanopsis echidnocarpa*	CASTEC	Fagaceae	Lower canopy	Nut	Animal, Gravity	881	0.9	291	518	72	High-plateau+; Low-slope−
11	*Castanopsis hystrix*	CASTHY	Fagaceae	Lower canopy	Nut	Animal, Gravity	244	0.3	32	146	66	High-plateau+; Low-slope−
12	*Castanopsis indica*	CASTIN	Fagaceae	Lower canopy	Nut	Animal, Gravity	351	0.4	166	132	53	Valley+; High-plateau−
13	*Baccaurea ramiflora*	BACCRA	Euphorbiaceae	Understory	Berry	Animal	3212	3.4	1814	1365	33	N
14	*Pittosporopsis kerrii*	PITTKE	Icacinaceae	Treelet	Drupe	Animal	20918	21.9	16439	4453	26	low-slope−; high-plateau+
15	*Mezzettiopsis creaghii*	MEZZCR	Annonaceae	Treelet	Berry	Animal	3300	3.5	1744	1514	42	Valley+; High-plateau−
16	*Nephelium chryseum*	NEPHCH	Sapindaceae	Treelet	Drupe	Animal	1098	1.1	713	343	42	high-slope+
16	*Phoebe lanceolata*	PHOELA	Lauraceae	Treelet	Drupe	Animal	2409	2.5	895	1496	18	Low-slope−; High-plateau+
17	*Saprosma ternata*	SAPRTE	Rubiaceae	Treelet	Drupe	Animal	2698	2.8	2332	345	21	Low-slope+; High-plateau−
19	*Dichapetalum gelonioides*	DICHGE	Dichapetalaceae	Treelet	Drupe	Animal	1222	1.3	704	473	45	High plateau−
20	*Leea compactiflora*	LEEACO	Vitaceae	Treelet	Berry	Animal	1051	1.1	974	64	13	N
	Total						57644	60.4				

Topographical types include valley (slope<27.1°; elevation<764.87 m), low-slope (slope>27.1°; elevation<764.87 m), high-slope (slope≥27.1°, elevation≥764.87 m, convexity>0), high-gully (slope≥27.1°, elevation≥764.87 m, convexity<0), high-plateau (slope<27.1°, elevation≥764.87 m, convexity>0) and gap (with a total open area greater than 200 m^2^). “+” indicates positive correlation; “−” indicates negative correlation; “N” indicates neutral correlation.

### Data analysis

In order to investigate the species distribution patterns and how the species associations change across life stages, individuals of these species were classified into three life stages: saplings (1 to 5 cm DBH), poles (5 to D95_0.1_ cm DBH) and adults (>D95_0.1_ cm DBH). Here, D95_0.1_ is the 95th percentile of diameter of all trees ≧0.1×Dmax, and Dmax is the diameter of the thickest tree 8,24,25,26. For treelets (maximum DBH no more than 20 cm), individuals between 1 and 3 cm in diameter were classified as saplings, and poles included stem diameters between 3 and D95_0.1_ cm. These divisions roughly correspond to trees that are located in the understory, midstory, and the canopy of the forest [27].

Positive and negative associations of species with habitats were determined by torus-translation tests. The tests assess the similarity between the spatial structure of each focal species population and each habitat [28]. Habitats of the plot were identified by three physical parameters ( i.e., elevation, slope and convexity) in each of the 20×20 m quadrats. We divided the habitat into six types, namely: valley (slope<27.1°; elevation<764.87 m), low-slope (slope>27.1°; elevation<764.87 m), high-slope (slope≥27.1°, elevation≥764.87 m, convexity>0), high-gully (slope≥27.1°, elevation≥764.87 m, convexity<0), high-plateau (slope<27.1°, elevation≥764.87 m, convexity>0) and gap. Here we defined a gap as such if it has a total open area greater than 200 m^2^. For the tests of association, each simulated map was overlaid by the true distribution of trees. Evaluation of all randomly seeded maps (n = 1000) and all torus-translated maps (n = 4999) gave frequency distributions of relative-density estimates for each species in each of the six principal habitats, one set of distributions for the randomized habitats tests and one set for the torus-translation tests, respectively.

### Spatial pattern analysis

We used the pair-correlation function [12], [29] as summary statistic to quantify the spatial structure of the uni- and bivariate patterns. The pair correlation function *g*_11_(*r*) for univariate patterns of a given life stage of a species 1 can be defined based on the neighborhood density *O*_11_(*r*) = λ_1_ *g*_11_(*r*) which is the mean density of trees of species 1 within rings with radius *r* and width *dr* centered in the trees of species 1 [12] where λ_1_ is the intensity ( = number of species 1 trees in the plot/area of the plot). The pair correlation function is therefore the ratio of the observed mean density of trees in the rings to the expected mean density of trees in the rings. The pair correlation function is especially suitable for exploratory analysis because it isolates specific distance classes [12]–[14], [29]. The pair correlation function for bivariate patterns (i.e., composed of species 1 and species 2 trees) follows intuitively, the quantity *g*_12_(*r*) is the ratio of the observed mean density of species 2 trees in the rings around species 1 trees to the expected mean density of species 2 trees in these rings [12]. The corresponding neighborhood density function yields *O*_12_(*r*) = λ_2_ *g*_12_(*r*).

The univariate pair correlation function *g*_11_(*r*) can be used to find out if the distribution of a species is random, aggregated, or regular; and at which distances *r* these patterns occur. Under the null model of complete spatial randomness (CSR), where the points are independently and randomly distributed over the entire plot, the pair correlation function yields *g*_11_(*r*) = 1, under aggregation *g*_11_(*r*)>1, and under regularity *g*_11_(*r*)<1 [12]. However, this assessment is more complicated in case of environmental heterogeneity [30]. If the pattern contains areas with low point density, the local neighborhood density is larger than the expected density under CSR. As a consequence, spurious aggregation appears which may also obscure an existing small-scale regularity (i.e., virtual aggregation; [30]).

One approach to account for possible first-order effects resulting from larger-scale environmental heterogeneity is to use the heterogeneous Poisson process as null model [12], [31]. This null model is able to approximately factor out the effects of heterogeneity by displacing the points of the pattern only within local neighborhoods of radius *h*. As a consequence, it maintains the observed large-scale structure but removes potential non-random local spatial structures at distances *r* below *h* [31]. This allows for an assessment of potential (conditional second-order) interactions among points if they occur at scales which are smaller than the scales at which the environment varies (i.e., a separation of scales [32]). The occurrence of any point in a heterogeneous Poisson point process is independent of that of others, but the points are distributed in accordance with an intensity function λ(x, y) that varies with location (x ,y) [12], [29], [30]. We used non-parametric kernel estimate of the intensity function based on the Epanechnikov kernel [12], [30] with a bandwidth of *h* = 30 m and a spatial resolution of 2 m.

We used the bivariate (cross) pair correlation function *g*_12_(*r*) to study the bivariate species-species associations. Again, first-order effects (habitat preference) may confound conditional second-order effects (direct plant-plant interactions) [30]. To reveal significant conditional second-order interaction we approximately factored out first-order effects by using the heterogeneous Poisson null model described above and randomized the locations of the trees of the second species, but we kept the locations of the trees of the first species fixed [30]. The intensity function was therefore constructed based on the pattern of the second species. A bandwidth of *h* = 30 m and a spatial resolution of 2 m were also selected for all bivariate analyses. To avoid edge effects, edge correction of Donnelly [33], available for rectangular windows only, was used in the analysis. Note that we assessed the g_21_-functions also for each species combination because we cannot assume that interactions between species would be symmetric [30].

We used a Monte-Carlo approach to test for significant departures from the null models. Each of the 199 simulations of a point process underlying the null model generates a summary statistic; and simulation envelopes with α≈0.05 were calculated from the 5th highest and lowest values of the 199 simulations [29]. Significant departure from the null model occurred at distance *r* if the test statistic was outside the simulation envelopes. This approach allowed us to assess scale effects and to determine the type of significant effect. Thus, the univariate analysis indicated aggregation if the observed *g*_11_(*r*) was above the simulation envelopes and regularity if it was below. Conversely, the bivariate analysis indicated a positive interaction if the observed *g*_12_(*r*) was above the simulation envelopes and a negative interaction if it was below. However, to avoid spurious rejection of the null model due to simultaneous inference (i.e., we conducted for each distance *r* one test) we used a goodness-of-fit (GoF) to test for overall departures from the null model for a predefined distance interval [34]. In our study, we selected a distance interval of 0–30 m to assess overall departures from the null model. Significant deviations from the null model were only considered as such if the observed *p* value of the GoF test was smaller than 0.05. All analyses were performed using the *Programita* software [12].

## Results

### Species distributions and topographic habitats

**Figure 2:**
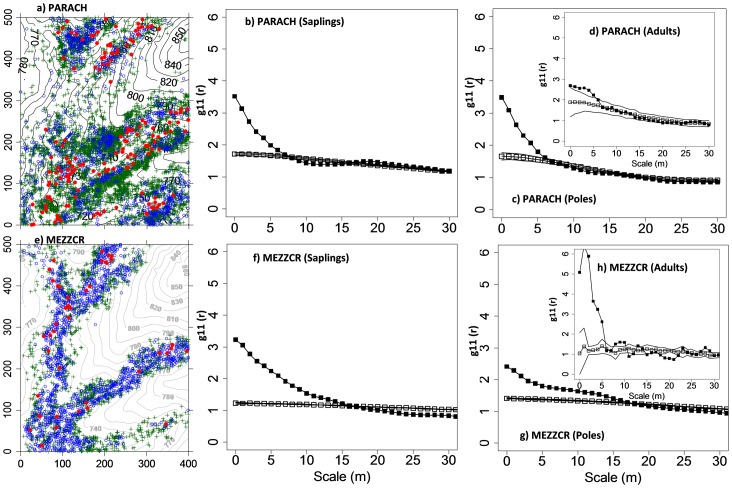
Examples of distribution maps and univariate patterns. Shown are the univariate g_11_ pair-correlation functions of the data in dependence on scale r (solid squares), and the expected g_11_(r) function under the heterogeneous Poisson null model (open squares) and the Monte Carlo simulation envelopes (solid lines) of the null models. Monte Carlo confidence was constructed at approximately 95% confidence level (199 simulations). See Table 1 for species codes. Green cross: saplings, blue open circle: poles, red solid circle: adults.

Distribution maps of the twenty tree species across three life stages are shown in Fig. S2. These maps show that trees are not uniformly distributed across the plot: some species are found in the gullies whereas others are found on the slope. The maps suggest that these twenty species have distribution patterns which are related to topography, especially for *P. chinensis* (Fig. S2a), *C. echidnocarpa* (Fig. S2j), *P. kerrii* (Fig. S2n), *M. creaghii* (Fig. S2o or Fig. 2e). Indeed, torus-translation tests [28] revealed that fourteen of the twenty species showed significant associations with at least one habitat type (Table 1). For example, *P. chinensis* (Fig. S2a) was negatively associated with the high-plateau and gaps; i.e., there were fewer individuals on the high-plateau or in gaps than expected. *P. tomentosa* (Fig. S2c) and *S. ternate* (Fig. S2r) were positively associated with low-slope, but negatively associated with high-slope and high-plateau, and *S. reticulata* (Fig. S2d), *C. indica* (Fig. S2l), were negatively associated with high-plateau. However, the test showed that there were no significant associations between the topographic habitats and the distributions of six species such as *S. tomentos* (Fig. S2b), *B. pendula* (Fig. S2e), *F. langkokensis* (Fig. S2h), *C. bejolghota* (Fig. S2i), *B. ramiflora* (Fig. S2l) and *L. compactiflora* (Fig. S2t).

### Species distribution patterns among life stages

Spatial pattern of each life stage of the twenty dominant tree species in the 20 ha plot were analyzed with the univariate pair correlation function. The saplings of all study species showed a strong small-scale aggregation at distances up to 10 m, whereas they followed at larger scales (10–30 m) mostly the heterogeneous Poisson null model (Fig. S3). Our results confirm the existence of separation of scales. Because the heterogeneous Poisson process conditions on the spatial structure for scales larger than 30 m, it is only able to indicate significant effects at scales smaller than 30 m. However, we found a strong additional signal of small-scale aggregation which disappears well below 30 m (mostly within 5 or 10 m). The regularity of *P. tomentosa* (Fig. S3c3) and *S. reticulata* (Fig. S3d3) is an artifact of the null model that appears if strongly localized aggregation appears. For poles, the spatial range and the intensity of small-scale aggregation declined compared to the saplings. For adults, though most species display a significant aggregation patterns at the adult stage (figure S3), the degree of spatial clumping deceases from saplings, to poles, to adults (Fig. S3). The loss of the strong clumping observed for juveniles with increasing life stage indicates density-dependent effects and is in agreement with our first guiding hypothesis.

### Species associations

Species associations among the twenty species across life stages were analyzed for scales 0–50 m by using bivariate analyses. In total there were 380 species combinations possible for each class of life stage; i.e., a total of 1140 species pairs were analyzed in this paper. For saplings, 162 (42.6%) of the 380 possible species pairs showed a significant and positive association at small neighborhoods below 5 m, but 146 species pairs (38.4%) showed negative associations at scales 0–5 m. The remaining 72 pairs (18.9%) showed no associations at scales 0–5 m. For poles and adults, positive small-scale associations occurred between 173 pairs (45.5%) and 112 pairs (29.5%), respectively; and 39.2% and 26.1% of the pairs showed negative associations. When comparing the development of the small scale-associations for increasing life stages we made the interesting observation that the number of significant association is higher for poles than for sapling but much lower for adult trees. This is indicated by an increase in the values of the pair correlation function at smaller distances that means that the relative neighborhood density increased.

**Figure 3:**
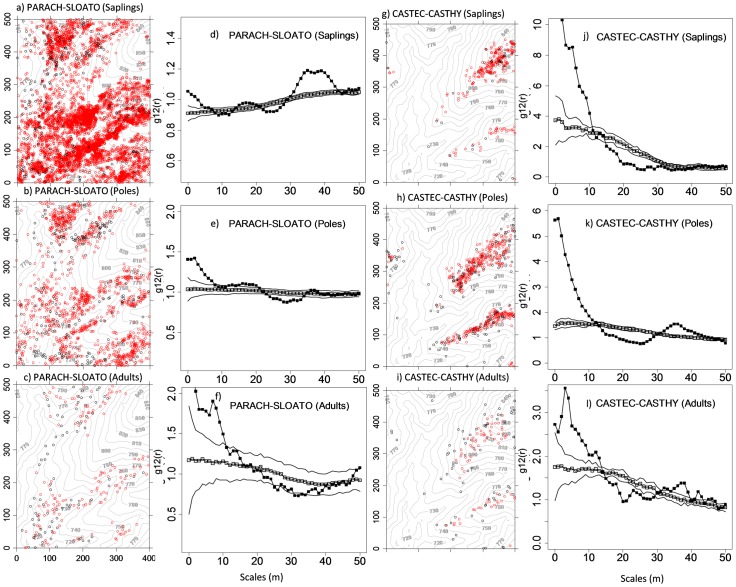
Examples of species associations in bivariate patterns. Shown are the bivariate g_12_ pair-correlation functions of the data in dependence on scale r (solid squares), the expected g_12_(r) function under the heterogeneous Poisson null model (open squares) and the simulation envelopes (solid lines) of the Monte Carlo simulations of the null model. Monte Carlo confidence was constructed at approximately 95% confidence level (199 simulations). See Table 1 for species codes. Red open circle is the first species, and black solid circle is the second species.

**Figure 4:**
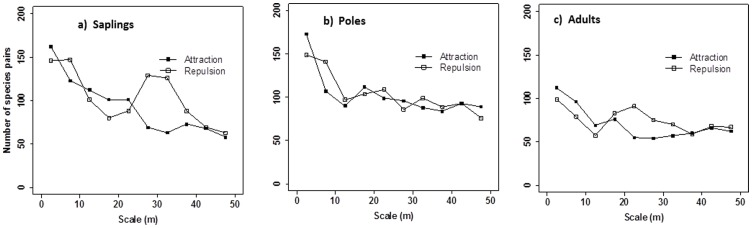
Number of significant attraction and repulsion for all species at the three life stages at scales 0–50 m. a) saplings, b) poles, c) adults.

Generally, the larger scale (>5 m) associations of all three life-stages did not show very strong evidence of species interactions. Figure 3 shows some examples of these bivariate analyses across life stages and all bivariate analyses can be found in Table S1. The strongly heterogeneous “banded” pattern of the species *P. chinensis* creates somewhat erratic pair correlation functions for bivariate patterns (e.g., Fig. 3d–f), but the important message here is the positive small-scale association at distances below 10 m. This indicates that individuals of the species *S. tomentosa* are more frequently located within neighborhoods of *P. chinensis* than expected by their overall distribution pattern (i.e., the intensity function). In order to investigate the change of the species associations across life stages, we plotted the number of species pairs against scales across three life stages (Fig. 4). We found for all three life stages a high proportion of positive small-scale (i.e., <10 m) associations. (Fig. 4). Surprisingly, this pattern was largely independent of life stage. However, the large-scale (i.e., >20 m) effects result mostly from the highly heterogeneous pattern of the species *P. chinensis* which showed a type of banded distribution pattern which creates the positive or negative larger scale association (e.g., Figs. 3d,e).

## Discussion

In this study we investigated how the univariate and bivariate spatial patterns of twenty dominant tree species changed in a tropical seasonal rain forest with life stage. We found strong small-scale aggregation of saplings, and for all life stages a high proportion of positive interspecific small-scale associations. Especially the positive association among the adult trees is somewhat surprising because one would expect that this pattern should disappear due to competition when trees grow to adult size. Thus we have the interesting situation to observe clear self thinning (i.e., loss of the small-scale aggregation with increasing size), but at the same time maintenance of the positive associations among some species. This pattern is compatible with simultaneous action of negative density dependence and a herd protection mechanism and/or facilitative interactions which favor survival of trees with more heterospecific neighbors. In the following we discuss our results in more detail.

### Sapling clustering

The twenty dominant species had a large number of juveniles which showed small-scale aggregation and were mostly located close to the adults (Fig. S2). Aggregated spatial distributions are commonly observed in naturally regenerating forests [35]–[36]. The present study provides evidence of clumping for the dominant twenty species in a dipterocarp forest in China. However, the degree of aggregation varied with species, life stages, and spatial scale. Aggregated distributions may result for example from limited seed dispersal [37], animal mediated clump dispersal [38], or habitat heterogeneity [39]–[41]. The topography of our site was very diverse, with an elevation ranging from 709 to 869 m above sea level and three perennial creeks that joined together in the south-eastern corner of the plot. Fourteen of the studied species showed a distribution pattern related to topography. This is confirmed by the species habitat (torus translation) test. However, the positive or negative associations to the topographic habitats are more likely to cause larger-scale aggregation which was approximately factored out by the heterogeneous Poisson null model. Thus, other factors may be responsible for the strong small-scale clustering of saplings. For example, we found especially strong small-scale clustering for saplings of the two species *P. chinensis* and *S. reticulate* (Fig. S2). Although *P. chinensis* produces winged seeds, the seeds are relatively heavy. Hence, *P. chinensis* is wind- and gravity-dispersed and as a consequence, nearly 60–70% of the seeds fall within a circle of 1–8 m near the conspecific adults [42] thereby causing the strongly aggregated distribution pattern at small scales. The species *S. tomentosa* produce capsules and when the fruits are ripe, seeds are launched upwards and dispersed ballistically with limited dispersal distance. The species *P. tomentosa*, *S. reticulata* produce drupes and are dispersed by animals. The species of *C. echidnocarpa*, *C. hystrix* and *C. indica* produce nuts and are dispersed by *Rattus tanezumi*, but the distance dispersed by *Rattus* is no more than 10 m [43]. This phenomenon is interesting because animal dispersal is known to be the most efficient dispersal mode in tropical forest [44]. However, both species *B. pendula* (with a mass about 220 g) and *G. cowa* (with a mass more than 70 g) have large seeds and are dispersed by gravity [45]. For *G. cowa*, there is the highest density of seedlings within a circle of 5–7 m near the conspecific adults [46]. Thus, for these tree species limited seed dispersal is likely to be responsible for the observed aggregated distribution pattern at small scales.

The spatial patterns of our study species lost their strong clumping from the transition from juveniles to adults and approached random patterns as found in previous studies [35], [47]. This indicates that density-dependent mortality of the offspring is ubiquitous in our plot [8]. However, self-thinning especially in tropical forests does not need to lead to regular distributions of tree species, but just random patterns may emerge [48]. Overall, our results on univariate species patterns of trees agree with our first hypothesis and support the presence of Janzen-Connell effects.

### Positive species associations

To investigate our second guiding hypothesis on species herd protection, we analyzed potential species interactions among twenty dominant species across three life stages. In order to disentangle the effect of species interactions (second-order effects) and environment (first-order effects) on the species association, we used heterogeneous Poisson null models, accounting for possible environmental heterogeneity, to reveal significant bivariate second-order interactions (repulsion, attraction). Our study revealed marked findings. One of the most interesting results is that second-order effects were strong; overall, 81% of all species pairs showed significant second-order effects (plant-plant interactions). Another interesting result is that positive associations between species made up a high proportion at small neighbourhoods of up to five meters. Interestingly, the highest proportion (45.5%) of positive associations occurred in the poles stage but it declined only to 29.5% in adults. The positive species interaction in the immediate neighbourhood of the youngest life-stage is compatible with the presence of species herd protection. With its emphasis on biotic interactions, this hypothesis is an extension of the classical Janzen-Connell hypothesis and states that increased heterospecific crowding results in fewer encounters between a host and its species-specific pests and pathogens. Thus, survival should increase with the density of heterospecifics, even if conspecific density remains constant [7], [49]. This hypothesis has been proposed to explain, for example, the high tree species diversity on Barro Colorado Island [6]. In addition, species interaction did likely not depend on phylogeny or taxonomy, for example *C. echidnocarpa* was positively associated with *C. hystrix* at scale 0–10 m (Fig. 3j-i), however *C. hystrix* was negatively associated with *C. indica* at scale 0–5m (Table S1), though these three species belong to the same genus. Only in 72 pairings of saplings showed no significant effects at small scales (0–5 m).

Somewhat unexpectedly we also observed positive associations between adult species. Given the larger size of adults and the observed (univariate) self thinning one would expect rather independence or negative interspecific associations. One explanation for the observed positive associations is that this pattern is still a signal from the species herd protection imprinted during earlier life stages. An alternative, non exclusive explanation is that the positive association could also be the result of facilitation. For example, *P. chinensis* is a big-statured species of the dipterocarpaceae with a huge umbrella crown which can grow up to 60 m in height. In the tropical seasonal rain forest of Xishuangbanna, the large trees of *P. chinensis* usually dominate the emergent layer and build up a relatively humid microenvironment favorable to the life of plants, especially in the dry season [15]. In other words, *P. chinensis* can facilitate the survival of other plants. We found that the associations among *P. chinensis* adults and adults of three other upper canopy species in our plot showed significant attraction (second-order effects) at small scales 0–5 m.

Previous research of tropical tree species associations were carried out in a Sri Lankan Dipterocarp forest, and the results showed that only 5% of common tree species pairs showed significant second-order effects (species interactions) [30]. However, in tropical seasonal rain forest in Xishuangbanna, 80% of the species pairs showed significant second-order effects (including both positive and negative association) at small scale 0–5 m. Although we do not know why there is such a big difference between the two dipterocarp forest, at least we can infer that mechanisms of tree species coexistence in dipterocarp tropical seasonal rainforest of China are totally different from dipterocarp tropical forest in Sri Lankan.

In conclusion, our results show that the degree of spatial clumping in the twenty dominant species decreases from saplings, to poles, to adults. The reduction of spatial aggregation with life stages is indirect evidence of Janzen–Connell spacing effects. Species distribution maps and the torus translation test indicate some presence of habitat associations and niche assembly. The positive spatial associations among the tree species at small spatial scales suggest operation of species herd protection but may also be indicative of facilitative interactions. Overall, these results suggest that Janzen–Connell spacing effects, habitat association, limited dispersal and species herd protection may contribute to shape the spatial patterns of the tree species in the tropical rainforest in Xishuangbanban, southwest China.

## Supporting Information

Figure S1 **Habitat of the 20-ha permanent plot of tropical seasonal rain forest in China.** Valley: slope<27.1°, elevation<764.87 m; Low-slope: slope>27.1°, elevation<764.87 m; High-slope: slope≥27.1°, elevation≥764.87 m, convexity>0; High-gully: slope≥27.1°, elevation≥764.87 m, convexity<0; High-plateau: slope<27.1°, elevation≥764.87 m, convexity>0; Gap: with a total open area greater than 200 m^2^.
(TIF)

Figure S2 **Distribution maps of the twenty dominant species across life stages in the 20-ha plot of tropical seasonal rainforest.** See Table 1 for species codes. Green cross: saplings, blue open circle: poles, red solid circle: adults.
(TIF)

Figure S3 **Univariate patterns of the twenty dominant species across life stages in the 20-ha plot of tropical seasonal rainforest.** Shown are the univariate g_11_ pair-correlation functions of the data in dependence on scale r (solid squares), and the expected g_11_(r) function under the heterogeneous Poisson null model (open squares) and the Monte Carlo simulation envelopes (solid lines) of the null models. Monte Carlo confidence was constructed at approximately 95% confidence level (199 simulations). See Table 1 for species codes.
(TIF)

Table S1 **Spatial associations of twenty dominant species across life stages in 20-ha permanent plot of tropical seasonal rain forest in China.** The bivariate statistic of the pair-correlation function was used to analyze the spatial associations among five canopy species under the heterogeneous Poison null model. “p” stands for positive association, “r” stands for no spatial association (randomness), and “n”for negative association. Monte Carlo simulation envelopes were constructed at the approximate 95% confidence level. See Table 1 for species codes.
(DOC)
