# Correction: Spatial Distribution and Interspecific Associations of Tree Species in a Tropical Seasonal Rain Forest of China

**DOI:** 10.1371/annotation/974531b0-9da4-4575-b3d1-955b0163fde0

**Published:** 2012-11-09

**Authors:** Guoyu Lan, Stephan Getzin, Thorsten Wiegand, Yuehua Hu, Guishui Xie, Hua Zhu, Min Cao

There is an error in the Funding section. The correct funding information is as follows: This project was supported by a grant from the National Science Foundation of China (31061160188-03) and a grant from the National Science & Technology Pillar Program of China (2008BAC39B02). SG was supported by the European Research Council Advanced Grant “SpatioDiversity” (grant number 233066). The funders had no role in study design, data collection and analysis, decision to publish, or preparation of the manuscript. 

