# Supplementary figures and images for: Spatial Distribution and Interspecific Associations of Tree Species in a Tropical Seasonal Rain Forest of China
Source: PLoS One. 2012 Sep 28;7(9):e46074. doi: 10.1371/journal.pone.0046074 (PMC3460976; doi:10.1371/journal.pone.0046074)

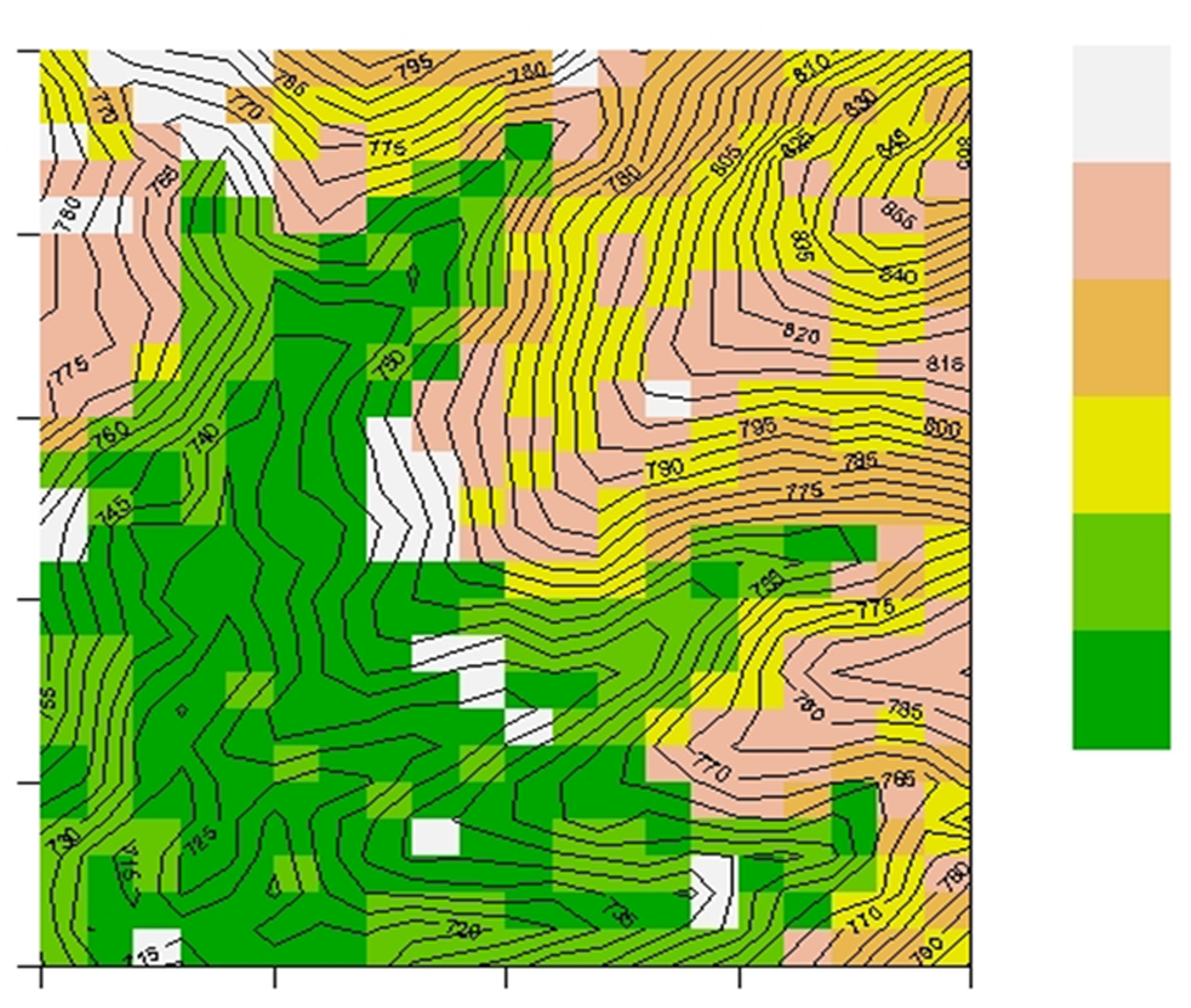

Supplement: Figure S1 — Habitat of the 20-ha permanent plot of tropical seasonal rain forest in China. Valley: slope<27.1°, elevation<764.87 m; Low-slope: slope>27.1°, elevation<764.87 m; High-slope: slope≥27.1°, elevation≥764.87 m, convexity>0; High-gully: slope≥27.1°, elevation≥764.87 m, convexity<0; High-plateau: slope<27.1°, elevation≥764.87 m, convexity>0; Gap: with a total open area greater than 200 m2. (TIF) [file pone.0046074.s001.tif]

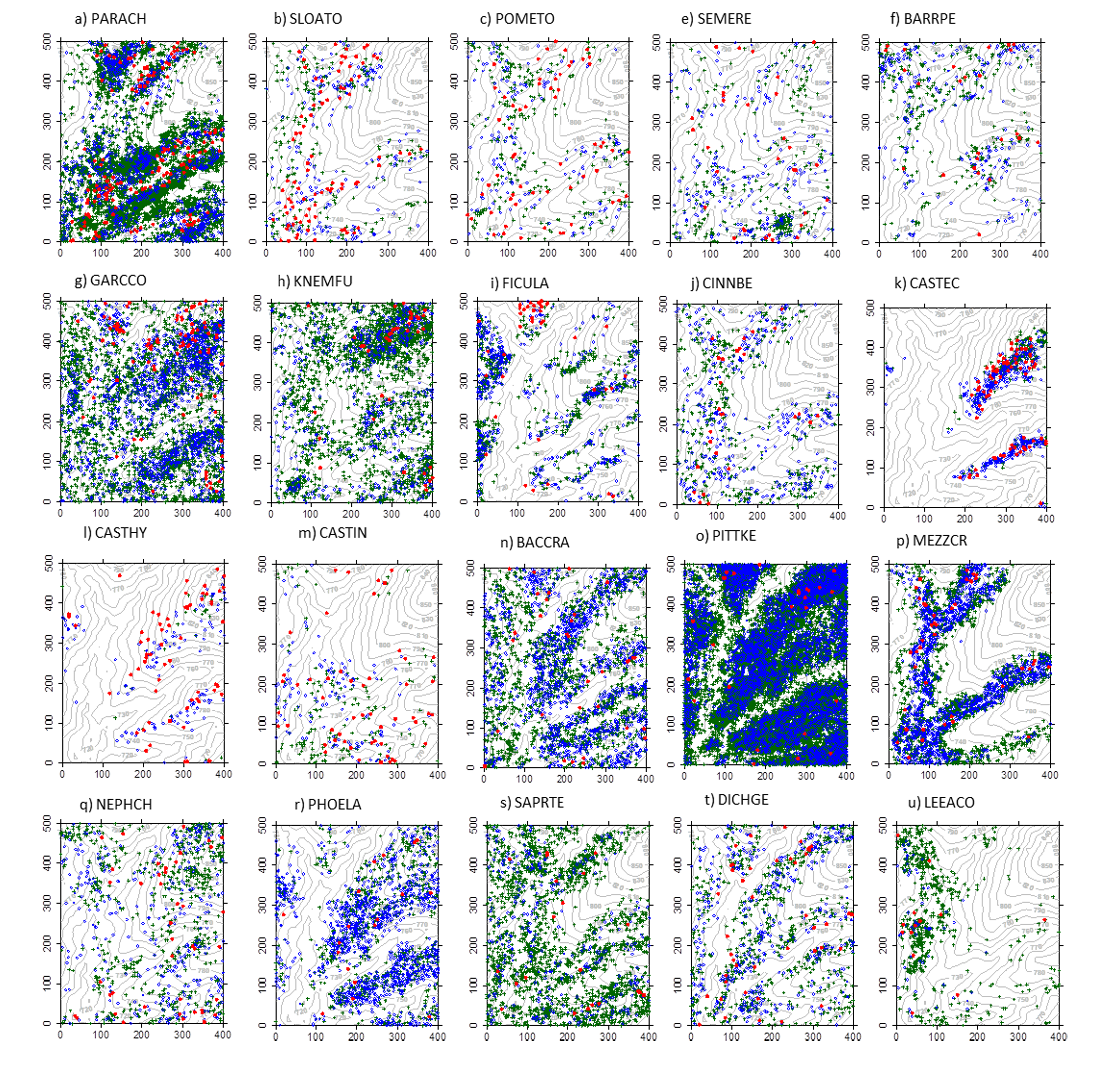

Supplement: Figure S2 — Distribution maps of the twenty dominant species across life stages in the 20-ha plot of tropical seasonal rainforest. See Table 1 for species codes. Green cross: saplings, blue open circle: poles, red solid circle: adults. (TIF) [file pone.0046074.s002.tif]

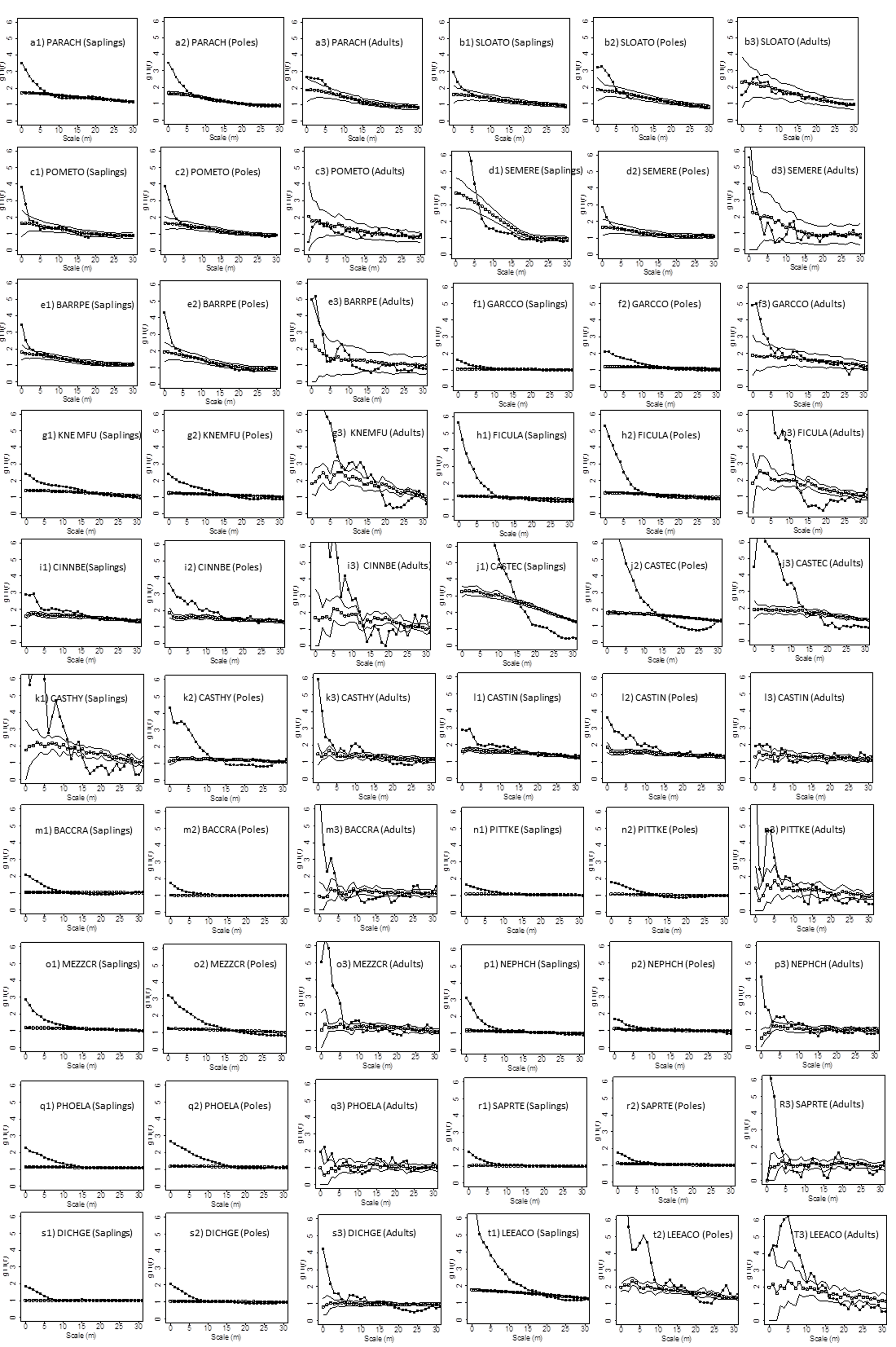

Supplement: Figure S3 — Univariate patterns of the twenty dominant species across life stages in the 20-ha plot of tropical seasonal rainforest. Shown are the univariate g11 pair-correlation functions of the data in dependence on scale r (solid squares), and the expected g11(r) function under the heterogeneous Poisson null model (open squares) and the Monte Carlo simulation envelopes (solid lines) of the null models. Monte Carlo confidence was constructed at approximately 95% confidence level (199 simulations). See Table 1 for species codes. (TIF) [file pone.0046074.s003.tif]
