# Supplementary material for: Spatial Distribution and Interspecific Associations of Tree Species in a Tropical Seasonal Rain Forest of China
Source: PLoS One. 2012 Sep 28;7(9):e46074. doi: 10.1371/journal.pone.0046074 (PMC3460976; doi:10.1371/journal.pone.0046074)
Supplement: Table S1 — Spatial associations of twenty dominant species across life stages in 20-ha permanent plot of tropical seasonal rain forest in China. The bivariate statistic of the pair-correlation function was used to analyze the spatial associations among five canopy species under the heterogeneous Poison null model. “p” stands for positive association, “r” stands for no spatial association (randomness), and “n”for negative association. Monte Carlo simulation envelopes were constructed at the approximate 95% confidence level. See Table 1 for species codes. (DOC) [file pone.0046074.s004.doc]

**Table S1: Spatial associations of twenty dominant species across life stages in 20-ha permanent plot of tropical seasonal rain forest in China. The bivariate statistic of the pair-correlation function was used to analyze the spatial associations among five canopy species under the heterogeneous Poison null model. “p” stands for positive association, “r” stands for no spatial association (randomness), and “n”for negative association. Monte Carlo simulation envelopes were constructed at the approximate 95% confidence level. See Table 1 for species codes.**

| **Species pairs** | **Scales (m)** | | | | | | | | | |
| --- | --- | --- | --- | --- | --- | --- | --- | --- | --- | --- |
| **Species A-Species B** | **0-5** | **6-10** | **11-15** | **16-20** | **21-25** | **26-30** | **31-35** | **36-40** | **41-45** | **46-50** |
| **Saplings** |  |  |  |  |  |  |  |  |  |  |
| BACCRA-BARRPE | n | n | r | r | r | r | n | n | n | r |
| BACCRA-CASTEC | n | n | p | p | r | n | n | r | r | r |
| BACCRA-CASTHY | n | n | r | p | p | r | n | r | p | r |
| BACCRA-CASTIN | r | r | r | r | r | r | r | r | r | r |
| BACCRA-CINNBE | n | r | r | p | p | r | r | r | r | r |
| BACCRA-DICHGE | p | r | r | r | r | r | r | r | r | r |
| BACCRAE-FICULA | p | n | n | r | r | p | r | r | r | r |
| BACCRA-GARCCO | p | p | p | n | r | r | r | r | p | p |
| BACCRA-KNEMFU | r | r | r | r | r | r | n | n | n | r |
| BACCRA-LEEACO | n | n | r | p | p | p | p | r | r | r |
| BACCRA-MEZZCR | n | n | p | p | p | p | r | r | r | n |
| BACCRA-NEPHCH | n | n | r | r | r | n | n | n | n | n |
| BACCRA-PARACH | p | r | p | p | p | p | p | p | p | r |
| BACCRA-PHOELA | p | r | r | r | r | r | r | r | r | r |
| BACCRA-PITTKE | p | p | r | r | r | r | r | r | r | r |
| BACCRA-POMETO | p | p | p | r | r | r | r | r | r | r |
| BACCRA-SAPRTE | p | p | p | p | p | p | p | p | n | n |
| BACCRA-SEMERE | r | p | p | p | r | n | n | r | r | r |
| BACCRA-SLOATO | r | n | r | r | r | r | r | r | n | r |
| BARRPE-BACCRA | n | n | n | p | p | p | p | n | n | n |
| BARRPE-CASTEC | n | n | n | p | p | p | n | n | n | r |
| BARRPE-CASTHY | n | n | n | r | p | r | n | n | n | n |
| BARRPE-CASTIN | p | p | n | n | n | n | r | r | r | n |
| BARRPE-CINNBE | r | r | n | r | r | p | p | r | r | r |
| BARRPE-DICHGE | p | r | r | r | r | n | n | n | n | r |
| BARRPE-FICULA | n | r | r | r | r | r | r | r | r | r |
| BARRPE-GARCCO | n | n | n | r | p | r | r | r | n | n |
| BARRPE-KNEMFU | p | p | n | n | n | r | r | p | p | r |
| BARRPE-LEEACO | p | p | r | n | n | n | r | p | p | p |
| BARRPE-MEZZCR | p | p | p | r | n | n | n | n | p | p |
| BARRPE-NEPHCH | p | r | n | n | r | n | n | n | r | r |
| BARRPE-PARACH | n | n | n | n | n | n | n | n | n | p |
| BARRPE-PHOELA | n | n | n | p | p | p | p | n | n | n |
| BARRPE-PITTKE | n | n | n | p | p | p | n | n | n | n |
| BARRPE-POMETO | r | r | r | r | r | r | r | r | r | r |
| BARRPE-SAPRTE | n | n | n | n | n | n | n | n | r | r |
| BARRPE-SEMERE | r | r | r | - | r | r | r | r | r | r |
| BARRPE-SLOATO | p | p | r | r | r | r | r | r | r | r |
| CASTEC-BACCRA | n | n | r | p | n | n | r | p | p | p |
| CASTEC-BARRPE | n | n | n | p | p | p | n | n | n | r |
| CASTEC-CASTHY | p | p | r | n | n | r | r | r | r | r |
| CASTEC-CASTIN | n | n | n | r | r | n | n | n | n | r |
| CASTEC-CINNBE | p | p | p | r | r | n | n | n | r | r |
| CASTEC-DICHGE | n | n | p | p | p | n | n | r | r | p |
| CASTECE-FICULA | n | r | p | p | r | r | n | n | n | n |
| CASTEC-GARCCO | p | p | p | n | n | n | n | r | p | p |
| CASTEC-KNEMFU | p | p | p | p | p | n | n | n | n | n |
| CASTEC-LEEACO | n | n | n | n | n | r | n | n | n | r |
| CASTEC-MEZZCR | n | n | n | r | p | p | p | p | r | r |
| CASTEC-NEPHCH | p | p | r | r | n | n | r | r | r | r |
| CASTEC-PARACH | n | n | n | n | n | n | n | p | p | p |
| CASTEC-PHOELA | p | p | p | n | n | n | r | p | p | p |
| CASTEC-PITTKE | n | r | p | p | n | n | n | p | p | p |
| CASTEC-POMETO | n | n | n | n | r | p | p | r | r | p |
| CASTEC-SAPRTE | n | n | n | p | p | n | n | n | n | r |
| CASTEC-SEMERE | p | r | r | n | r | r | n | n | n | n |
| CASTEC-SLOATO | n | n | n | r | r | r | p | r | r | r |
| CASTHY-BACCRA | n | n | r | r | r | n | n | p | p | p |
| CASTHY-BARRPE | n | n | n | r | p | p | n | n | n | n |
| CASTHY-CASTEC | p | p | r | n | n | n | n | r | r | r |
| CASTHY-CASTIN | n | r | n | r | p | r | n | r | n | n |
| CASTHY-CINNBE | p | p | r | r | r | n | n | r | n | r |
| CASTHY-DICHGE | n | n | r | p | r | r | n | r | r | p |
| CASTHYE-FICULA | n | r | r | r | r | n | n | n | n | n |
| CASTHY-GARCCO | p | p | r | n | n | n | r | r | p | r |
| CASTHY-KNEMFU | p | p | p | p | r | n | n | n | n | r |
| CASTHY-LEEACO | n | n | r | r | p | r | r | n | r | r |
| CASTHY-MEZZCR | n | n | n | r | p | p | p | p | r | r |
| CASTHY-NEPHCH | p | p | r | r | r | n | r | r | r | r |
| CASTHY-PARACH | r | n | n | p | p | n | n | p | p | p |
| CASTHY-PHOELA | p | p | p | n | n | r | r | p | p | r |
| CASTHY-PITTKE | p | p | p | r | n | n | n | r | p | p |
| CASTHY-POMETO | n | n | n | r | p | r | p | r | p | r |
| CASTHY-SAPRTE | n | n | n | r | p | r | n | r | r | r |
| CASTHY-SEMERE | n | r | p | p | n | n | r | n | n | r |
| CASTHY-SLOATO | n | n | n | r | p | r | p | r | r | r |
| CASTIN-BACCRA | p | r | r | r | r | r | r | p | r | r |
| CASTIN-BARRPE | p | p | r | r | n | n | r | r | r | n |
| CASTIN-CASTEC | n | n | n | p | p | r | n | n | n | n |
| CASTIN-CASTHY | n | r | n | p | p | r | r | n | n | n |
| CASTIN-CINNBE | r | r | r | r | r | p | p | p | r | n |
| CASTIN-DICHGE | p | n | r | r | r | r | r | r | r | r |
| CASTINE-FICULA | r | n | n | r | r | p | p | p | r | r |
| CASTIN-GARCCO | n | n | r | r | p | p | r | r | r | r |
| CASTIN-KNEMFU | p | p | r | n | n | n | r | r | r | r |
| CASTIN-LEEACO | r | r | r | r | r | n | r | r | p | r |
| CASTIN-MEZZCR | p | p | p | p | p | r | r | r | r | n |
| CASTIN-NEPHCH | p | n | r | r | r | r | n | n | n | n |
| CASTIN-PARACH | p | p | p | p | r | r | r | r | r | n |
| CASTIN-PHOELA | n | n | r | p | p | p | p | r | r | r |
| CASTIN-PITTKE | r | n | p | p | p | r | r | r | r | r |
| CASTIN-POMETO | p | p | r | r | r | p | r | r | r | r |
| CASTIN-SAPRTE | r | r | r | n | r | r | r | r | r | r |
| CASTIN-SEMERE | p | p | r | r | r | r | p | p | p | n |
| CASTIN-SLOATO | p | r | r | r | r | r | p | r | r | r |
| CINNBE-BACCRA | n | n | r | r | r | r | r | r | r | r |
| CINNBE-BARRPE | r | r | n | r | r | p | p | r | r | n |
| CINNBE-CASTEC | p | p | p | r | n | n | n | n | r | r |
| CINNBE-CASTHY | p | p | r | r | r | n | r | r | r | r |
| CINNBE-CASTIN | r | r | r | n | n | r | r | r | n | n |
| CINNBE-DICHGE | p | n | r | p | r | r | r | r | r | r |
| CINNBE-FICULA | p | n | n | p | p | r | r | r | r | r |
| CINNBE-GARCCO | p | p | r | r | r | n | n | n | p | p |
| CINNBE-KNEMFU | p | p | p | n | n | n | n | n | n | n |
| CINNBE-LEEACO | p | p | p | r | r | n | r | r | r | r |
| CINNBE-MEZZCR | n | n | r | r | p | p | p | p | p | n |
| CINNBE-NEPHCH | p | r | r | r | n | n | r | r | r | r |
| CINNBE-PARACH | n | n | n | n | n | n | p | p | p | p |
| CINNBE-PHOELA | p | p | p | r | r | r | r | p | p | p |
| CINNBE-PITTKE | r | r | r | r | r | n | n | r | r | r |
| CINNBE-POMETO | p | p | r | r | r | r | r | r | r | r |
| CINNBE-SAPRTE | r | r | p | r | n | r | p | r | r | r |
| CINNBE-SEMERE | p | p | p | p | n | n | n | r | r | n |
| CINNBE-SLOATO | r | r | p | r | n | r | r | r | r | r |
| DICHGE-BACCRA | p | r | r | r | r | r | r | r | r | r |
| DICHGE-BARRPE | r | r | r | r | r | r | r | r | r | r |
| DICHGE-CASTEC | n | n | r | p | p | r | r | p | p | p |
| DICHGE-CASTHY | n | n | r | p | p | r | n | p | p | p |
| DICHGE-CASTIN | p | r | r | r | r | r | r | r | r | r |
| DICHGE-CINNBE | p | n | r | p | r | r | r | r | r | r |
| DICHGE-FICULA | p | n | n | r | r | p | p | r | r | r |
| DICHGE-GARCCO | n | n | n | r | p | p | r | r | r | r |
| DICHGE-KNEMFU | p | r | r | r | r | r | r | p | p | p |
| DICHGE-LEEACO | n | n | r | p | p | r | r | p | r | r |
| DICHGE-MEZZCR | r | p | p | p | r | r | r | r | r | r |
| DICHGE-NEPHCH | r | r | r | r | r | n | n | r | r | r |
| DICHGE-PARACH | p | r | r | p | p | p | r | n | r | p |
| DICHGE-PHOELA | p | r | r | r | p | p | r | r | r | r |
| DICHGE-PITTKE | p | r | r | r | r | r | r | r | r | r |
| DICHGE-POMETO | r | r | r | p | r | r | r | r | r | r |
| DICHGE-SAPRTE | p | r | r | r | r | r | r | r | r | r |
| DICHGE-SEMERE | n | n | r | r | p | r | r | p | r | n |
| DICHGE-SLOATO | r | r | p | r | r | r | r | r | r | r |
| FICULA-BACCRA | p | n | n | r | r | p | p | p | r | n |
| FICULA-BARRPE | r | r | p | r | r | r | r | r | p | r |
| FICULA-CASTEC | n | p | p | p | r | n | n | n | n | n |
| FICULA-CASTHY | n | p | r | r | p | r | r | n | r | r |
| FICULA-CASTIN | r | n | n | n | n | p | r | r | n | r |
| FICULA-CINNBE | p | n | r | p | p | r | r | r | r | r |
| FICULA-DICHGE | p | n | n | r | r | p | p | r | r | p |
| FICULA-GARCCO | n | p | r | r | p | p | n | n | r | r |
| FICULA-KNEMFU | n | n | r | r | p | r | r | r | r | r |
| FICULA-LEEACO | n | n | p | p | p | r | n | p | p | r |
| FICULA-MEZZCR | n | n | p | p | r | r | p | r | r | r |
| FICULA-NEPHCH | r | r | r | r | r | n | r | r | r | r |
| FICULA-PARACH | n | n | n | n | n | n | n | p | p | p |
| FICULA-PHOELA | p | r | r | r | r | r | r | r | r | r |
| FICULA-PITTKE | p | n | n | n | n | r | p | p | r | r |
| FICULA-POMETO | n | n | r | p | p | p | p | p | n | n |
| FICULA-SAPRTE | p | n | n | n | n | p | p | p | r | r |
| FICULA-SEMERE | r | n | r | p | n | n | n | n | n | n |
| FICULA-SLOATO | n | n | n | n | p | p | p | p | p | p |
| GARCCO-BACCRA | p | p | r | r | r | r | r | p | p | r |
| GARCCO-BARRPE | n | n | r | r | p | r | r | n | n | n |
| GARCCO-CASTEC | p | p | p | n | n | n | n | n | r | r |
| GARCCO-CASTHY | p | p | p | n | n | n | r | r | r | r |
| GARCCO-CASTIN | n | n | n | n | r | r | r | r | n | n |
| GARCCO-CINNBE | p | p | p | r | r | n | n | n | p | p |
| GARCCO-DICHGE | n | n | n | r | p | r | r | r | r | r |
| GARCCO-FICULA | p | p | n | n | p | r | n | n | r | r |
| GARCCO-KNEMFU | n | n | p | p | r | n | n | n | n | r |
| GARCCO-LEEACO | r | r | p | r | r | n | r | r | r | p |
| GARCCO-MEZZCR | n | n | n | p | p | p | p | r | r | r |
| GARCCO-NEPHCH | r | r | r | r | r | n | n | n | n | n |
| GARCCO-PARACH | n | r | r | p | p | p | p | p | p | r |
| GARCCO-PHOELA | p | p | p | n | n | r | p | p | p | r |
| GARCCO-PITTKE | p | p | n | n | r | r | r | r | r | r |
| GARCCO-POMETO | n | n | r | r | r | r | r | r | r | r |
| GARCCO-SAPRTE | n | n | r | p | p | p | r | r | r | r |
| GARCCO-SEMERE | n | p | p | r | r | n | n | n | n | r |
| GARCCO-SLOATO | n | n | p | p | r | r | r | r | r | r |
| PITTKE-CASTIN | r | n | r | r | r | r | r | r | r | r |
| KNEMFU-BACCRA | n | n | r | r | r | r | r | r | r | r |
| KNEMFU-BARRPE | p | r | r | r | r | r | r | p | p | r |
| KNEMFU-CASTEC | p | p | p | p | p | n | n | n | n | r |
| KNEMFU-CASTHY | r | p | p | p | r | n | n | n | n | r |
| KNEMFU-CASTIN | p | r | r | n | n | n | n | r | r | r |
| KNEMFU-CINNBE | p | p | p | r | r | r | r | r | r | r |
| KNEMFU-DICHGE | p | p | r | n | n | n | n | n | r | r |
| KNEMFU-FICULA | n | n | n | r | r | r | r | r | r | n |
| KNEMFU-GARCCO | n | r | p | p | p | r | n | n | n | n |
| KNEMFU-LEEACO | r | r | r | n | n | n | n | r | r | r |
| KNEMFU-MEZZCR | p | p | n | n | n | n | p | p | p | p |
| KNEMFU-NEPHCH | p | p | p | r | n | n | n | n | r | r |
| KNEMFU-PARACH | n | n | n | n | n | n | n | p | p | p |
| KNEMFU-PHOELA | n | n | p | p | p | r | n | n | r | r |
| KNEMFU-PITTKE | p | r | r | r | r | r | p | r | r | r |
| KNEMFU-POMETO | p | p | n | n | n | n | r | p | p | p |
| KNEMFU-SAPRTE | p | p | r | n | n | n | n | r | r | r |
| KNEMFU-SEMERE | p | p | r | n | r | r | n | n | p | n |
| KNEMFU-SLOATO | p | p | n | n | n | n | r | r | r | r |
| LEEACO-BACCRA | n | r | r | r | r | r | n | n | r | r |
| LEEACO-BARRPE | p | p | p | n | n | n | r | p | p | p |
| LEEACO-CASTEC | n | n | n | n | n | n | n | n | n | n |
| LEEACO-CASTHY | n | n | n | r | p | r | n | n | r | r |
| LEEACO-CASTIN | n | p | p | r | n | n | n | p | p | r |
| LEEACO-CINNBE | p | p | p | n | n | n | n | r | r | r |
| LEEACO-DICHGE | n | n | p | r | r | r | n | p | r | r |
| LEEACO-FICULA | n | p | p | p | r | n | n | r | p | r |
| LEEACO-GARCCO | r | r | p | r | n | n | n | n | r | p |
| LEEACO-KNEMFU | r | r | r | n | n | n | n | p | p | p |
| LEEACO-MEZZCR | p | p | r | n | n | p | p | p | r | r |
| LEEACO-NEPHCH | r | n | r | r | n | n | n | n | n | r |
| LEEACO-PARACH | n | n | n | n | n | n | r | p | p | r |
| LEEACO-PHOELA | n | n | n | p | p | p | r | r | r | r |
| LEEACO-PITTKE | n | n | r | p | p | n | n | n | r | p |
| LEEACO-POMETO | r | r | p | p | n | n | r | r | r | r |
| LEEACO-SAPRTE | n | p | p | r | n | n | n | r | p | p |
| LEEACO-SEMERE | r | r | r | n | r | n | n | n | n | p |
| LEEACO-SLOATO | p | p | p | n | n | n | r | p | p | r |
| MEZZCR-BACCRA | n | n | p | p | p | r | r | r | r | r |
| MEZZCR-BARRPE | p | p | p | r | n | n | n | r | p | p |
| MEZZCR-CASTEC | n | n | n | r | p | p | p | n | n | n |
| MEZZCR-CASTHY | n | n | r | r | r | r | r | p | n | r |
| MEZZCR-CASTIN | p | p | p | r | r | r | r | r | r | r |
| MEZZCR-CINNBE | n | n | r | r | r | p | p | r | r | n |
| MEZZCR-DICHGE | r | p | p | p | r | r | r | r | r | r |
| MEZZCRE-FICULA | n | n | n | r | r | r | p | r | r | p |
| MEZZCR-GARCCO | n | n | n | p | p | p | r | r | r | r |
| MEZZCR-KNEMFU | p | p | r | n | n | n | p | p | r | r |
| MEZZCR-LEEACO | r | p | r | n | r | p | p | r | r | n |
| MEZZCR-NEPHCH | p | r | n | n | r | r | n | n | r | r |
| MEZZCR-PARACH | n | p | p | p | n | n | r | p | p | r |
| MEZZCR-PHOELA | n | n | p | p | p | p | p | r | r | r |
| MEZZCR-PITTKE | n | n | p | p | r | r | r | r | r | r |
| MEZZCR-POMETO | p | p | p | r | r | r | r | r | r | r |
| MEZZCR-SAPRTE | p | p | p | r | r | r | r | r | r | r |
| MEZZCR-SEMERE | p | p | n | n | p | p | n | r | r | r |
| MEZZCR-SLOATO | p | p | p | r | n | n | n | n | r | r |
| NEPHCH-BACCRA | n | n | r | r | r | r | r | r | r | r |
| NEPHCH-BARRPE | p | r | r | r | r | r | r | r | r | r |
| NEPHCH-CASTEC | p | p | p | r | n | n | n | r | n | n |
| NEPHCH-CASTHY | p | p | r | r | r | n | n | r | r | r |
| NEPHCH-CASTIN | p | n | r | r | n | r | n | n | n | r |
| NEPHCH-CINNBE | p | p | r | r | r | n | r | r | r | r |
| NEPHCH-DICHGE | r | r | r | r | r | r | r | p | p | r |
| NEPHCH-FICULA | r | n | r | r | r | n | r | r | r | r |
| NEPHCH-GARCCO | r | r | p | r | r | n | n | r | r | r |
| NEPHCH-KNEMFU | p | p | p | p | p | n | n | n | r | r |
| NEPHCH-LEEACO | r | n | p | r | n | n | n | n | r | p |
| NEPHCH-MEZZCR | p | p | n | n | r | r | r | r | p | p |
| NEPHCH-PARACH | r | r | r | r | r | r | r | p | r | r |
| NEPHCH-PHOELA | r | r | p | r | p | p | p | r | r | r |
| NEPHCH-PITTKE | r | r | p | r | r | n | r | r | p | r |
| NEPHCH-POMETO | p | p | r | n | n | n | r | r | r | p |
| NEPHCH-SAPRTE | p | r | n | r | r | r | r | r | r | r |
| NEPHCH-SEMERE | p | p | r | n | r | r | r | r | r | r |
| NEPHCH-SLOATO | r | r | r | r | r | r | r | r | r | p |
| PARACH-BACCRA | p | r | r | p | p | r | r | r | r | r |
| PARACH-BARRPE | n | n | n | n | n | n | n | n | n | n |
| PARACH-CASTEC | n | n | n | n | n | n | n | p | p | p |
| PARACH-CASTHY | r | r | r | r | p | r | r | r | r | p |
| PARACH-CASTIN | p | p | r | r | r | r | r | r | r | r |
| PARACH-CINNBE | n | n | n | n | n | n | p | p | r | p |
| PARACH-DICHGE | p | r | r | p | p | p | n | r | p | p |
| PARACH-FICULA | n | n | n | n | n | n | n | p | p | p |
| PARACH-GARCCO | n | n | n | r | p | p | p | p | r | r |
| PARACH-KNEMFU | n | n | n | n | n | n | n | n | p | p |
| PARACH-LEEACO | n | n | n | n | r | p | p | p | p | r |
| PARACH-MEZZCR | n | p | p | p | r | n | r | r | r | r |
| PARACH-NEPHCH | r | r | r | r | r | r | r | r | r | r |
| PARACH-PHOELA | p | r | n | n | p | p | r | r | r | r |
| PARACH-PITTKE | p | r | r | r | p | p | r | r | r | r |
| PARACH-POMETO | r | r | r | r | r | r | r | r | r | r |
| PARACH-SAPRTE | p | n | p | p | r | r | r | r | r | r |
| PARACH-SEMERE | n | r | r | r | r | n | p | p | r | r |
| PARACH-SLOATO | r | r | r | r | r | r | r | r | r | r |
| PHOELA-BACCRA | p | r | r | r | r | r | r | r | r | r |
| PHOELA-BARRPE | n | n | n | p | p | p | n | n | n | n |
| PHOELA-CASTEC | p | p | p | r | n | n | n | p | p | p |
| PHOELA-CASTHY | p | p | p | r | n | n | n | r | r | r |
| PHOELA-CASTIN | n | n | n | r | r | r | r | n | n | n |
| PHOELA-CINNBE | p | p | p | r | r | n | n | r | p | p |
| PHOELA-DICHGE | p | r | n | r | p | r | r | r | r | r |
| PHOELA-FICULA | p | r | n | r | r | r | r | r | r | r |
| PHOELA-GARCCO | p | p | r | n | n | r | r | r | r | r |
| PHOELA-KNEMFU | n | n | p | p | p | n | n | n | n | r |
| PHOELA-LEEACO | n | n | r | p | p | p | r | r | r | r |
| PHOELA-MEZZCR | n | n | p | p | p | p | r | r | r | r |
| PHOELA-NEPHCH | r | n | r | n | r | n | n | n | n | n |
| PHOELA-PARACH | p | p | n | r | p | p | r | r | r | r |
| PHOELA-PITTKE | p | p | n | n | n | r | r | r | r | r |
| PHOELA-POMETO | n | n | r | r | p | r | r | r | r | p |
| PHOELA-SAPRTE | n | n | n | r | p | r | n | n | r | r |
| PHOELA-SEMERE | n | r | p | r | r | r | n | p | p | n |
| PHOELA-SLOATO | n | n | r | r | r | p | r | r | r | r |
| PITTKE-BACCRA | p | p | r | r | r | r | r | r | r | r |
| PITTKE-BARRPE | n | n | n | p | p | r | r | n | n | n |
| PITTKE-CASTEC | n | p | p | p | r | n | n | n | p | p |
| PITTKE-CASTHY | r | p | p | r | r | n | n | r | r | r |
| PITTKE-CINNBE | r | r | r | r | r | r | r | r | r | r |
| PITTKE-DICHGE | p | r | n | r | r | r | r | r | r | r |
| PITTKEE-FICULA | p | n | n | n | n | r | r | r | r | r |
| PITTKE-GARCCO | p | p | r | r | r | r | r | r | r | r |
| PITTKE-KNEMFU | p | p | p | r | r | n | n | n | n | r |
| PITTKE-LEEACO | n | n | n | p | p | p | n | n | n | r |
| PITTKE-MEZZCR | n | n | p | p | r | r | p | p | p | p |
| PITTKE-NEPHCH | r | r | r | r | n | n | n | n | n | n |
| PITTKE-PARACH | p | p | p | p | p | p | p | r | r | r |
| PITTKE-PHOELA | p | p | r | n | r | p | p | r | r | r |
| PITTKE-POMETO | n | r | p | p | r | r | r | r | r | r |
| PITTKE-SAPRTE | r | r | r | r | r | r | r | r | r | r |
| PITTKE-SEMERE | n | p | p | p | r | n | n | r | r | n |
| PITTKE-SLOATO | n | n | p | p | r | r | r | r | r | r |
| POMETO-BACCRA | n | p | r | r | p | r | r | r | r | p |
| POMETO-BARRPE | r | r | r | r | r | r | r | r | r | r |
| POMETO-CASTEC | n | n | n | n | r | p | r | n | n | p |
| POMETO-CASTHY | n | n | n | r | p | r | r | r | n | r |
| POMETO-CASTIN | p | r | r | r | p | r | r | r | r | r |
| POMETO-CINNBE | p | p | r | r | r | r | r | r | r | r |
| POMETO-DICHGE | r | p | p | r | r | r | n | r | r | r |
| POMETO-FICULA | n | n | n | p | p | p | p | p | r | r |
| POMETO-GARCCO | n | n | p | p | r | r | r | r | r | r |
| POMETO-KNEMFU | p | p | n | n | n | n | r | r | r | r |
| POMETO-LEEACO | r | r | p | p | r | n | r | r | n | r |
| POMETO-MEZZCR | p | p | p | r | r | r | r | r | r | r |
| POMETO-NEPHCH | p | r | r | n | n | n | n | n | r | r |
| POMETO-PARACH | p | p | p | r | r | p | p | p | p | r |
| POMETO-PHOELA | n | n | r | r | p | p | p | r | r | r |
| POMETO-PITTKE | n | n | r | r | r | r | r | r | r | r |
| POMETO-SAPRTE | p | p | p | r | r | r | r | r | r | r |
| POMETO-SEMERE | p | p | r | r | r | n | r | p | p | r |
| POMETO-SLOATO | p | p | r | r | r | r | r | r | r | r |
| SAPRTE-BACCRA | n | n | p | p | p | p | p | p | p | p |
| SAPRTE-BARRPE | r | n | n | n | n | n | n | n | p | r |
| SAPRTE-CASTEC | n | n | n | p | p | n | n | n | n | t |
| SAPRTE-CASTHY | n | n | n | p | p | n | r | r | r | n |
| SAPRTE-CASTIN | p | r | r | r | r | r | p | r | r | r |
| SAPRTE-CINNBE | r | r | r | r | n | n | r | r | r | r |
| SAPRTE-DICHGE | p | r | r | r | r | r | r | r | r | r |
| SAPRTE-FICULA | p | n | n | r | r | r | r | r | r | r |
| SAPRTE-GARCCO | n | n | n | p | p | r | n | n | n | r |
| SAPRTE-KNEMFU | p | p | r | n | n | n | n | r | r | r |
| SAPRTE-LEEACO | n | p | p | p | r | n | n | r | r | r |
| SAPRTE-MEZZCR | p | p | p | r | r | r | r | r | r | r |
| SAPRTE-NEPHCH | p | n | n | r | n | n | n | n | r | r |
| SAPRTE-PARACH | p | n | p | p | r | r | r | r | r | r |
| SAPRTE-PHOELA | n | n | r | p | p | p | p | r | r | r |
| SAPRTE-PITTKE | r | r | r | p | r | r | r | r | n | n |
| SAPRTE-POMETO | p | r | n | n | n | n | n | n | r | r |
| SAPRTE-SEMERE | p | r | r | r | r | r | r | r | r | r |
| SAPRTE-SLOATO | p | r | r | r | r | r | r | r | r | r |
| SEMERE-BACCRA | r | r | p | p | r | n | n | p | p | p |
| SEMERE-BARRPE | r | r | r | r | r | r | r | r | r | r |
| SEMERE-CASTEC | p | p | r | n | p | p | n | n | n | n |
| SEMERE-CASTHY | r | r | p | p | r | r | r | r | r | r |
| SEMERE-CASTIN | r | r | r | r | r | r | r | p | p | n |
| SEMERE-CINNBE | p | p | p | r | n | r | r | r | r | n |
| SEMERE-DICHGE | n | n | r | r | p | r | r | p | r | n |
| SEMERE-FICULA | n | n | n | p | n | n | n | n | n | n |
| SEMERE-GARCCO | n | r | p | p | r | r | n | n | n | r |
| SEMERE-KNEMFU | p | p | r | r | r | r | r | p | r | n |
| SEMERE-LEEACO | r | r | r | n | r | n | n | n | n | p |
| SEMERE-MEZZCR | p | p | n | n | p | p | p | r | r | r |
| SEMERE-NEPHCH | p | r | r | r | r | r | r | r | r | r |
| SEMERE-PARACH | n | n | p | p | n | p | p | p | r | n |
| SEMERE-PHOELA | n | n | p | r | r | r | r | p | r | n |
| SEMERE-PITTKE | n | p | p | p | r | n | n | r | p | n |
| SEMERE-POMETO | r | r | r | r | r | r | r | p | r | r |
| SEMERE-SAPRTE | p | p | n | n | p | r | r | r | r | r |
| SEMERE-SLOATO | p | r | r | r | r | r | r | r | r | r |
| SLOATO-BACCRA | n | n | r | p | p | p | p | r | r | r |
| SLOATO-BARRPE | p | p | r | r | r | r | r | r | r | r |
| SLOATO-CASTEC | n | n | n | r | p | r | p | r | r | r |
| SLOATO-CASTHY | n | n | n | r | p | r | p | r | r | r |
| SLOATO-CASTIN | r | r | n | r | r | r | r | r | r | r |
| SLOATO-CINNBE | r | r | p | r | r | r | p | p | r | r |
| SLOATO-DICHGE | r | r | r | r | r | n | r | r | r | r |
| SLOATO-FICULA | n | n | p | r | r | r | p | r | r | r |
| SLOATO-GARCCO | n | n | r | p | r | r | r | r | r | r |
| SLOATO-KNEMFU | p | p | n | n | n | n | r | r | r | r |
| SLOATO-LEEACO | p | p | p | r | r | r | r | r | r | r |
| SLOATO-MEZZCR | p | p | p | r | r | r | n | n | r | r |
| SLOATO-NEPHCH | r | r | r | r | r | r | r | r | r | r |
| SLOATO-PARACH | p | r | r | r | r | n | p | p | r | r |
| SLOATO-PHOELA | n | n | r | r | r | p | r | r | r | r |
| SLOATO-PITTKE | n | n | p | p | p | r | r | r | r | r |
| SLOATO-POMETO | p | p | r | r | r | r | r | r | r | r |
| SLOATO-SAPRTE | p | p | r | r | r | r | r | r | r | r |
| SLOATO-SEMERE | p | r | r | r | r | r | r | r | r | r |
| **Poles** |  |  |  |  |  |  |  |  |  |  |
| BACCRA-BARRPE | r | n | r | r | r | r | r | r | n | n |
| BACCRA-CASTEC | n | n | p | p | p | r | n | n | p | p |
| BACCRA-CASTHY | r | p | p | r | r | r | r | r | p | p |
| BACCRA-CASTIN | p | p | r | n | r | r | r | r | r | r |
| BACCRA-CINNBE | p | r | r | r | r | p | r | r | n | r |
| BACCRA-DICHGE | p | p | n | n | r | p | p | p | r | n |
| BACCRAE-FICULA | p | r | n | n | r | p | p | p | n | n |
| BACCRA-GARCCO | r | p | p | r | p | p | r | n | p | p |
| BACCRA-KNEMFU | p | r | r | r | r | r | r | r | r | n |
| BACCRA-LEEACO | r | n | n | r | r | p | r | p | p | p |
| BACCRA-MEZZCR | n | n | p | p | p | r | r | r | r | n |
| BACCRA-NEPHCH | p | n | r | r | r | n | r | r | n | n |
| BACCRA-PARACH | p | p | r | r | r | p | r | r | r | r |
| BACCRA-PHOELA | p | r | r | r | r | r | r | r | r | r |
| BACCRA-PITTKE | p | p | r | n | n | p | p | r | n | r |
| BACCRA-POMETO | n | r | p | p | r | r | r | r | r | r |
| BACCRA-SAPRTE | r | r | n | r | r | p | p | p | r | r |
| BACCRA-SEMERE | p | r | r | r | r | r | r | r | r | n |
| BACCRA-SLOATO | r | r | r | r | r | p | p | r | r | r |
| BARRPE-BACCRA | r | n | n | r | p | p | p | p | r | n |
| BARRPE-CASTEC | n | n | p | p | p | p | n | n | n | n |
| BARRPE-CASTHY | n | n | p | p | p | p | n | n | n | r |
| BARRPE-CASTIN | r | r | n | r | r | r | r | r | r | p |
| BARRPE-CINNBE | n | n | p | p | p | p | n | n | n | r |
| BARRPE-DICHGE | p | r | n | r | r | r | n | r | r | n |
| BARRPE-FICULA | r | r | r | r | p | r | p | p | p | p |
| BARRPE-GARCCO | n | n | r | p | p | p | r | n | n | r |
| BARRPE-KNEMFU | p | p | p | n | n | n | n | p | p | p |
| BARRPE-LEEACO | p | p | r | r | n | n | r | r | p | r |
| BARRPE-MEZZCR | p | p | p | n | n | n | r | p | p | p |
| BARRPE-NEPHCH | p | p | r | r | n | n | n | r | r | r |
| BARRPE-PARACH | n | n | n | r | r | r | n | r | r | n |
| BARRPE-PHOELA | n | n | r | p | p | p | r | n | n | r |
| BARRPE-PITTKE | n | n | r | p | p | r | n | n | n | n |
| BARRPE-POMETO | p | p | r | r | r | r | r | r | r | r |
| BARRPE-SAPRTE | n | n | n | n | n | n | r | r | r | n |
| BARRPE-SEMERE | r | r | r | r | r | r | r | r | r | r |
| BARRPE-SLOATO | r | r | r | r | r | r | r | p | p | r |
| CASTEC-BACCRA | n | n | p | p | p | r | n | r | p | p |
| CASTEC-BARRPE | n | n | p | p | p | n | n | n | n | r |
| CASTEC-CASTHY | p | p | r | n | n | n | p | p | p | r |
| CASTEC-CASTIN | n | r | r | r | r | r | r | r | r | r |
| CASTEC-CINNBE | r | p | r | n | n | n | n | p | p | p |
| CASTEC-DICHGE | n | n | r | p | p | p | r | r | n | p |
| CASTECE-FICULA | n | p | p | n | n | n | n | n | p | r |
| CASTEC-GARCCO | p | p | p | n | n | n | p | p | p | r |
| CASTEC-KNEMFU | n | n | p | p | p | p | n | n | n | r |
| CASTEC-LEEACO | n | n | n | r | n | n | n | r | p | p |
| CASTEC-MEZZCR | n | n | n | p | p | p | p | n | n | n |
| CASTEC-NEPHCH | n | p | r | r | r | r | n | r | n | r |
| CASTEC-PARACH | n | n | n | n | p | p | p | p | p | r |
| CASTEC-PHOELA | p | p | r | n | n | n | p | p | p | n |
| CASTEC-PITTKE | p | p | r | n | n | n | n | p | p | p |
| CASTEC-POMETO | n | n | r | p | p | r | r | n | n | r |
| CASTEC-SAPRTE | n | n | n | n | n | n | n | p | p | p |
| CASTEC-SEMERE | n | n | r | r | r | r | p | n | n | r |
| CASTEC-SLOATO | n | n | n | p | p | p | r | n | n | n |
| CASTHY-BACCRA | n | p | r | r | r | r | r | r | p | p |
| CASTHY-BARRPE | n | n | p | p | p | n | n | n | n | r |
| CASTHY-CASTEC | p | p | r | n | n | p | p | p | p | r |
| CASTHY-CASTIN | r | r | r | r | r | r | p | p | p | r |
| CASTHY-CINNBE | p | p | r | n | n | r | r | r | p | p |
| CASTHY-DICHGE | n | r | r | r | r | p | p | p | r | r |
| CASTHYE-FICULA | p | p | r | n | n | n | n | r | r | n |
| CASTHY-GARCCO | p | p | n | n | n | n | p | p | p | r |
| CASTHY-KNEMFU | n | n | p | p | p | p | n | n | n | p |
| CASTHY-LEEACO | n | r | r | r | r | r | n | r | r | p |
| CASTHY-MEZZCR | n | n | r | p | p | p | n | n | n | r |
| CASTHY-NEPHCH | n | r | p | r | r | r | r | n | n | r |
| CASTHY-PARACH | n | n | n | n | p | p | p | p | p | p |
| CASTHY-PHOELA | p | p | n | n | n | r | p | p | p | n |
| CASTHY-PITTKE | p | p | n | n | n | n | p | p | p | r |
| CASTHY-POMETO | n | n | r | p | p | p | n | n | n | n |
| CASTHY-SAPRTE | n | n | r | p | p | r | r | r | r | r |
| CASTHY-SEMERE | n | r | r | r | r | r | r | n | r | p |
| CASTHY-SLOATO | n | n | n | p | p | p | r | n | n | n |
| CASTIN-BACCRA | p | p | r | p | r | r | r | r | r | r |
| CASTIN-BARRPE | n | n | n | r | r | r | n | n | n | p |
| CASTIN-CASTEC | n | r | n | r | p | p | r | p | p | p |
| CASTIN-CASTHY | r | r | r | r | r | r | p | p | p | r |
| CASTIN-CINNBE | p | r | r | r | r | r | r | r | r | r |
| CASTIN-DICHGE | p | r | r | r | r | p | p | p | r | r |
| CASTINE-FICULA | p | r | n | n | n | r | p | p | r | n |
| CASTIN-GARCCO | r | r | r | r | r | r | r | r | r | p |
| CASTIN-KNEMFU | n | n | r | r | r | r | r | r | p | r |
| CASTIN-LEEACO | n | r | p | p | p | r | n | r | r | r |
| CASTIN-MEZZCR | n | n | p | p | p | p | p | r | r | r |
| CASTIN-NEPHCH | r | n | r | n | r | r | r | r | r | r |
| CASTIN-PARACH | p | r | p | p | r | r | r | r | r | r |
| CASTIN-PHOELA | p | p | r | n | r | r | p | p | p | r |
| CASTIN-PITTKE | p | r | r | n | n | r | r | r | n | n |
| CASTIN-POMETO | n | r | p | p | r | n | n | r | r | r |
| CASTIN-SAPRTE | r | r | p | p | r | r | n | r | r | p |
| CASTIN-SEMERE | p | p | p | r | r | r | r | r | p | r |
| CASTIN-SLOATO | r | r | r | r | r | r | p | p | p | p |
| CINNBE-BACCRA | p | r | r | r | r | r | r | n | n | r |
| CINNBE-BARRPE | n | n | r | r | r | r | n | n | n | r |
| CINNBE-CASTEC | n | p | p | n | n | n | n | p | p | p |
| CINNBE-CASTHY | r | p | r | n | n | r | r | p | p | p |
| CINNBE-CASTIN | p | r | r | r | r | r | r | r | p | p |
| CINNBE-DICHGE | p | n | r | r | r | r | r | r | r | n |
| CINNBE-FICULA | p | r | n | p | r | r | r | r | n | n |
| CINNBE-GARCCO | p | p | r | n | n | n | n | r | p | p |
| CINNBE-KNEMFU | r | n | n | r | p | p | r | n | n | n |
| CINNBE-LEEACO | p | r | r | p | r | r | r | r | r | n |
| CINNBE-MEZZCR | n | n | p | p | p | p | r | n | n | n |
| CINNBE-NEPHCH | p | r | r | r | r | n | n | r | n | n |
| CINNBE-PARACH | p | n | n | r | p | p | p | r | n | r |
| CINNBE-PHOELA | p | p | r | r | n | r | p | p | p | p |
| CINNBE-PITTKE | p | p | n | n | n | r | r | r | r | r |
| CINNBE-POMETO | n | r | p | p | r | r | n | r | r | r |
| CINNBE-SAPRTE | p | r | r | p | r | r | r | r | n | n |
| CINNBE-SEMERE | p | r | p | r | r | r | r | r | r | n |
| CINNBE-SLOATO | n | n | p | p | p | p | r | n | n | n |
| DICHGE-BACCRA | p | p | r | p | n | p | p | p | r | n |
| DICHGE-BARRPE | p | p | n | r | r | r | n | r | r | n |
| DICHGE-CASTEC | n | n | r | p | r | r | r | r | r | p |
| DICHGE-CASTHY | n | r | r | r | r | r | r | r | r | r |
| DICHGE-CASTIN | p | r | r | r | r | p | r | r | n | n |
| DICHGE-CINNBE | p | r | n | r | r | r | r | p | r | n |
| DICHGE-FICULA | p | n | n | n | n | r | p | r | n | n |
| DICHGE-GARCCO | n | n | n | r | r | r | r | r | r | r |
| DICHGE-KNEMFU | p | r | r | r | n | r | r | r | r | r |
| DICHGE-LEEACO | r | r | n | r | r | p | p | n | r | n |
| DICHGE-MEZZCR | n | p | p | p | r | n | n | n | r | r |
| DICHGE-NEPHCH | p | r | r | r | r | r | r | r | r | r |
| DICHGE-PARACH | p | r | r | n | r | r | r | r | r | r |
| DICHGE-PHOELA | r | r | n | r | r | p | p | p | r | r |
| DICHGE-PITTKE | p | p | n | n | n | r | p | r | r | r |
| DICHGE-POMETO | n | r | p | r | r | r | n | n | r | r |
| DICHGE-SAPRTE | p | r | r | r | r | r | r | r | r | r |
| DICHGE-SEMERE | p | r | r | r | r | r | r | r | r | n |
| DICHGE-SLOATO | p | p | p | r | n | n | r | n | r | r |
| FICULA-BACCRA | p | n | n | n | n | p | p | r | n | n |
| FICULA-BARRPE | r | r | r | r | r | r | p | p | p | p |
| FICULA-CASTEC | n | p | p | n | r | r | n | n | p | p |
| FICULA-CASTHY | p | p | r | n | n | r | r | p | p | r |
| FICULA-CASTIN | p | r | n | n | n | r | p | p | r | r |
| FICULA-CINNBE | p | r | n | r | p | r | r | r | n | n |
| FICULA-DICHGE | p | n | n | n | n | r | p | r | n | n |
| FICULA-GARCCO | p | p | r | n | r | p | n | n | r | r |
| FICULA-KNEMFU | r | n | n | r | r | r | n | n | n | r |
| FICULA-LEEACO | p | p | p | p | p | p | n | n | n | n |
| FICULA-MEZZCR | n | n | p | p | p | r | r | r | r | p |
| FICULA-NEPHCH | p | n | r | r | r | r | r | r | r | r |
| FICULA-PARACH | n | n | n | n | n | p | p | p | p | p |
| FICULA-PHOELA | p | r | r | r | r | n | p | r | r | r |
| FICULA-PITTKE | p | n | n | n | n | p | p | r | n | r |
| FICULA-POMETO | n | r | p | p | r | r | r | r | n | r |
| FICULA-SAPRTE | r | n | r | p | p | r | n | n | n | r |
| FICULA-SEMERE | r | n | r | r | r | n | r | r | r | r |
| FICULA-SLOATO | n | n | r | p | p | p | r | r | r | r |
| GARCCO-BACCRA | r | r | r | r | p | p | r | r | r | r |
| GARCCO-BARRPE | n | n | n | p | p | r | n | n | n | n |
| GARCCO-CASTEC | p | p | p | n | n | n | p | p | p | p |
| GARCCO-CASTHY | p | p | r | n | n | n | p | p | p | r |
| GARCCO-CASTIN | r | r | n | n | r | r | r | r | p | p |
| GARCCO-CINNBE | p | p | r | n | n | n | n | r | p | p |
| GARCCO-DICHGE | n | n | r | r | p | p | r | r | r | r |
| GARCCO-FICULA | n | r | n | n | r | r | n | n | n | n |
| GARCCO-KNEMFU | n | n | p | p | p | p | n | n | n | n |
| GARCCO-LEEACO | r | n | r | r | r | n | n | r | r | p |
| GARCCO-MEZZCR | n | n | n | p | p | p | p | p | r | p |
| GARCCO-NEPHCH | n | r | r | r | r | n | n | n | n | r |
| GARCCO-PARACH | r | p | r | r | r | r | r | p | p | p |
| GARCCO-PHOELA | p | p | r | n | n | p | p | p | p | p |
| GARCCO-PITTKE | p | p | n | n | n | r | r | r | p | p |
| GARCCO-POMETO | n | n | p | p | p | r | r | r | r | r |
| GARCCO-SAPRTE | n | n | p | p | p | n | n | r | r | p |
| GARCCO-SEMERE | n | r | r | r | r | r | r | r | p | r |
| GARCCO-SLOATO | n | n | n | p | p | p | p | n | n | n |
| PITTKE-CASTIN | p | r | r | n | n | r | r | r | r | p |
| KNEMFU-BACCRA | p | r | n | r | r | p | p | p | r | n |
| KNEMFU-BARRPE | p | p | p | n | n | n | n | p | p | r |
| KNEMFU-CASTEC | n | n | p | p | p | p | n | n | n | r |
| KNEMFU-CASTHY | n | n | n | p | p | p | n | n | n | p |
| KNEMFU-CASTIN | n | n | r | r | r | r | r | p | p | r |
| KNEMFU-CINNBE | r | n | r | r | r | r | r | r | r | r |
| KNEMFU-DICHGE | p | p | r | r | n | n | n | r | r | r |
| KNEMFU-FICULA | r | n | n | r | r | r | n | n | n | r |
| KNEMFU-GARCCO | n | n | p | p | p | p | n | n | n | r |
| KNEMFU-LEEACO | p | r | r | n | n | n | n | p | p | p |
| KNEMFU-MEZZCR | p | p | p | n | n | n | p | p | p | p |
| KNEMFU-NEPHCH | p | r | r | r | n | n | n | r | r | r |
| KNEMFU-PARACH | p | r | r | n | n | p | p | p | p | p |
| KNEMFU-PHOELA | n | n | r | p | p | p | r | n | n | r |
| KNEMFU-PITTKE | p | r | r | r | r | r | n | r | r | r |
| KNEMFU-POMETO | p | p | r | n | n | r | r | p | p | p |
| KNEMFU-SAPRTE | p | p | r | n | n | n | r | r | r | r |
| KNEMFU-SEMERE | p | p | r | n | n | r | r | r | r | r |
| KNEMFU-SLOATO | p | p | p | n | n | n | r | r | r | r |
| LEEACO-BACCRA | r | n | n | n | n | n | n | r | p | p |
| LEEACO-BARRPE | p | r | r | n | n | n | n | r | p | p |
| LEEACO-CASTEC | n | n | n | r | r | n | n | n | n | r |
| LEEACO-CASTHY | r | r | p | r | r | n | n | n | r | r |
| LEEACO-CASTIN | n | r | p | p | r | n | n | r | p | p |
| LEEACO-CINNBE | p | n | r | r | n | r | n | r | r | r |
| LEEACO-DICHGE | r | r | n | r | r | p | n | r | r | r |
| LEEACO-FICULA | p | p | p | p | p | r | n | n | r | r |
| LEEACO-GARCCO | r | n | r | n | n | n | n | n | r | r |
| LEEACO-KNEMFU | p | r | n | n | n | n | n | r | r | r |
| LEEACO-MEZZCR | n | r | r | n | n | r | p | p | r | r |
| LEEACO-NEPHCH | n | r | r | p | r | n | n | n | n | r |
| LEEACO-PARACH | n | n | n | n | r | r | r | r | p | r |
| LEEACO-PHOELA | n | n | n | p | p | r | r | r | p | p |
| LEEACO-PITTKE | n | n | n | r | r | n | n | n | r | p |
| LEEACO-POMETO | r | p | r | n | n | r | p | r | r | r |
| LEEACO-SAPRTE | p | p | r | n | n | r | p | r | r | r |
| LEEACO-SEMERE | n | r | r | r | n | n | r | r | r | r |
| LEEACO-SLOATO | n | n | n | n | n | r | r | p | p | p |
| MEZZCR-BACCRA | n | n | p | p | r | r | r | p | r | n |
| MEZZCR-BARRPE | p | p | r | n | n | n | r | r | p | p |
| MEZZCR-CASTEC | n | n | n | p | p | p | p | n | n | n |
| MEZZCR-CASTHY | n | n | p | p | p | p | r | n | n | n |
| MEZZCR-CASTIN | n | n | n | p | p | p | p | p | p | r |
| MEZZCR-CINNBE | n | n | p | p | p | r | r | r | n | n |
| MEZZCR-DICHGE | n | p | p | p | p | n | n | n | r | p |
| MEZZCRE-FICULA | n | n | r | p | r | r | r | r | r | p |
| MEZZCR-GARCCO | n | n | n | p | p | p | p | p | r | p |
| MEZZCR-KNEMFU | p | p | r | n | n | n | r | p | p | p |
| MEZZCR-LEEACO | n | p | p | r | n | r | p | p | n | n |
| MEZZCR-NEPHCH | n | r | r | n | r | r | r | r | r | p |
| MEZZCR-PARACH | n | p | p | n | n | n | r | r | r | r |
| MEZZCR-PHOELA | n | n | n | p | p | p | p | n | n | n |
| MEZZCR-PITTKE | n | n | p | p | p | r | r | r | r | n |
| MEZZCR-POMETO | p | p | r | n | n | n | n | r | r | p |
| MEZZCR-SAPRTE | p | p | p | r | r | r | r | r | r | r |
| MEZZCR-SEMERE | p | p | r | r | p | r | r | r | r | r |
| MEZZCR-SLOATO | p | p | p | p | n | n | n | r | r | r |
| NEPHCH-BACCRA | p | n | r | r | r | r | r | p | r | n |
| NEPHCH-BARRPE | p | p | r | r | n | n | n | n | n | r |
| NEPHCH-CASTEC | n | p | p | p | p | p | r | r | r | r |
| NEPHCH-CASTHY | n | p | p | r | p | p | r | r | r | r |
| NEPHCH-CASTIN | r | n | r | n | r | r | r | r | p | p |
| NEPHCH-CINNBE | p | r | r | p | r | n | r | r | r | n |
| NEPHCH-DICHGE | p | r | r | r | r | r | r | r | r | r |
| NEPHCH-FICULA | p | n | n | r | r | r | n | n | r | r |
| NEPHCH-GARCCO | n | p | p | p | p | r | r | n | r | r |
| NEPHCH-KNEMFU | p | p | p | r | n | n | n | n | r | r |
| NEPHCH-LEEACO | r | r | r | p | n | n | n | n | n | r |
| NEPHCH-MEZZCR | n | r | r | r | r | p | r | r | r | p |
| NEPHCH-PARACH | p | n | n | r | p | r | p | p | r | r |
| NEPHCH-PHOELA | r | n | r | p | p | p | p | p | p | r |
| NEPHCH-PITTKE | p | r | r | r | r | r | r | r | r | n |
| NEPHCH-POMETO | p | r | r | r | r | n | n | r | r | p |
| NEPHCH-SAPRTE | p | r | r | r | r | r | r | r | r | r |
| NEPHCH-SEMERE | p | r | r | r | r | r | r | r | r | r |
| NEPHCH-SLOATO | p | r | r | r | r | r | r | r | n | p |
| PARACH-BACCRA | p | r | r | r | r | p | r | r | r | r |
| PARACH-BARRPE | n | n | n | n | n | n | n | n | n | n |
| PARACH-CASTEC | n | n | n | n | n | n | n | p | p | p |
| PARACH-CASTHY | n | n | n | n | p | p | p | p | p | p |
| PARACH-CASTIN | r | r | p | p | r | r | r | r | p | p |
| PARACH-CINNBE | p | n | p | p | p | p | p | p | n | p |
| PARACH-DICHGE | p | r | n | n | r | r | r | p | r | r |
| PARACH-FICULA | n | n | n | n | n | n | p | p | p | p |
| PARACH-GARCCO | n | n | n | r | p | p | r | r | r | r |
| PARACH-KNEMFU | p | r | r | n | n | r | r | r | p | r |
| PARACH-LEEACO | n | n | n | r | p | p | p | p | p | n |
| PARACH-MEZZCR | n | p | p | p | n | n | r | r | r | r |
| PARACH-NEPHCH | p | n | n | r | r | n | r | r | r | r |
| PARACH-PHOELA | n | r | r | r | p | p | r | r | r | r |
| PARACH-PITTKE | p | n | n | n | r | r | r | r | r | r |
| PARACH-POMETO | p | p | p | r | r | n | r | r | r | r |
| PARACH-SAPRTE | p | r | r | p | r | r | r | r | r | r |
| PARACH-SEMERE | r | r | r | p | r | r | r | r | r | r |
| PARACH-SLOATO | p | r | p | r | r | r | r | r | r | r |
| PHOELA-BACCRA | p | r | r | r | r | r | r | r | r | r |
| PHOELA-BARRPE | n | n | r | p | p | r | n | n | n | n |
| PHOELA-CASTEC | p | p | p | n | n | n | p | p | p | n |
| PHOELA-CASTHY | p | p | n | n | n | n | p | p | p | n |
| PHOELA-CASTIN | p | p | r | n | r | r | p | p | r | r |
| PHOELA-CINNBE | p | r | r | n | n | r | r | p | p | p |
| PHOELA-DICHGE | p | r | n | n | r | r | r | r | r | r |
| PHOELA-FICULA | p | r | r | n | n | r | r | r | r | r |
| PHOELA-GARCCO | p | p | r | n | n | r | p | p | p | r |
| PHOELA-KNEMFU | n | n | n | p | p | r | n | n | n | n |
| PHOELA-LEEACO | n | n | n | r | r | n | n | n | p | p |
| PHOELA-MEZZCR | n | n | n | p | p | p | r | n | n | n |
| PHOELA-NEPHCH | r | n | r | r | r | r | r | n | n | r |
| PHOELA-PARACH | r | r | r | r | p | p | r | r | r | r |
| PHOELA-PITTKE | p | p | n | n | n | n | r | r | r | r |
| PHOELA-POMETO | n | n | r | p | p | p | n | n | r | r |
| PHOELA-SAPRTE | n | n | r | r | r | r | n | n | r | r |
| PHOELA-SEMERE | n | r | r | r | r | r | r | r | r | r |
| PHOELA-SLOATO | n | n | r | r | r | r | r | r | r | r |
| PITTKE-BACCRA | p | p | n | n | n | p | p | r | r | r |
| PITTKE-BARRPE | n | n | r | p | p | r | n | n | n | n |
| PITTKE-CASTEC | p | p | r | r | r | r | r | p | p | p |
| PITTKE-CASTHY | p | p | r | n | n | r | p | p | p | r |
| PITTKE-CINNBE | p | p | n | n | n | r | r | r | r | r |
| PITTKE-DICHGE | p | r | n | n | n | r | p | r | r | r |
| PITTKEE-FICULA | p | r | n | n | n | r | p | r | n | r |
| PITTKE-GARCCO | p | p | p | r | r | r | r | r | p | p |
| PITTKE-KNEMFU | p | r | n | r | r | r | r | r | r | r |
| PITTKE-LEEACO | n | n | n | p | p | p | n | n | r | r |
| PITTKE-MEZZCR | n | n | p | p | p | p | r | r | r | r |
| PITTKE-NEPHCH | p | r | r | r | r | r | r | r | n | n |
| PITTKE-PARACH | p | p | r | r | r | r | r | r | r | r |
| PITTKE-PHOELA | p | p | n | n | n | p | p | p | p | p |
| PITTKE-POMETO | n | n | p | p | r | n | n | r | r | r |
| PITTKE-SAPRTE | n | n | n | r | r | r | r | n | n | r |
| PITTKE-SEMERE | p | r | r | r | r | n | r | r | r | r |
| PITTKE-SLOATO | n | n | r | p | p | p | p | r | n | n |
| POMETO-BACCRA | n | n | p | p | p | p | p | p | p | n |
| POMETO-BARRPE | p | p | r | n | n | r | r | r | r | r |
| POMETO-CASTEC | n | n | n | p | p | p | p | n | n | p |
| POMETO-CASTHY | n | n | r | p | p | p | p | r | r | r |
| POMETO-CASTIN | n | r | p | p | p | r | r | p | p | p |
| POMETO-CINNBE | n | r | p | p | r | r | r | r | n | n |
| POMETO-DICHGE | r | p | p | r | n | n | n | r | p | p |
| POMETO-FICULA | n | n | r | p | p | r | r | r | n | r |
| POMETO-GARCCO | n | n | p | p | p | p | p | p | r | r |
| POMETO-KNEMFU | p | p | n | n | n | n | r | r | p | r |
| POMETO-LEEACO | r | r | r | r | r | r | p | r | r | n |
| POMETO-MEZZCR | p | p | r | n | n | r | r | r | p | p |
| POMETO-NEPHCH | p | r | r | n | r | n | n | n | r | r |
| POMETO-PARACH | p | p | p | r | r | n | r | p | r | r |
| POMETO-PHOELA | n | n | n | p | p | p | p | r | r | r |
| POMETO-PITTKE | n | n | p | p | r | n | p | r | r | n |
| POMETO-SAPRTE | p | p | n | n | n | r | r | r | r | r |
| POMETO-SEMERE | r | r | r | r | r | r | r | r | r | r |
| POMETO-SLOATO | p | p | r | r | r | r | r | r | p | r |
| SAPRTE-BACCRA | r | r | r | r | r | r | r | r | r | r |
| SAPRTE-BARRPE | r | r | n | n | n | n | n | r | r | r |
| SAPRTE-CASTEC | n | n | n | n | n | n | n | n | p | p |
| SAPRTE-CASTHY | n | n | r | p | p | r | n | n | n | r |
| SAPRTE-CASTIN | r | r | p | r | r | r | r | r | r | p |
| SAPRTE-CINNBE | p | r | r | p | r | r | r | r | n | r |
| SAPRTE-DICHGE | p | r | r | r | r | r | r | r | r | r |
| SAPRTE-FICULA | r | n | r | p | p | r | r | n | n | r |
| SAPRTE-GARCCO | n | n | p | p | p | r | n | n | n | p |
| SAPRTE-KNEMFU | p | p | r | n | n | r | r | r | r | r |
| SAPRTE-LEEACO | p | p | r | r | r | r | p | r | n | r |
| SAPRTE-MEZZCR | p | p | r | r | r | r | n | r | r | r |
| SAPRTE-NEPHCH | p | r | r | r | n | n | n | n | n | r |
| SAPRTE-PARACH | p | r | r | r | n | r | r | r | r | r |
| SAPRTE-PHOELA | n | n | r | p | p | p | p | p | r | r |
| SAPRTE-PITTKE | n | n | r | r | r | r | r | n | n | r |
| SAPRTE-POMETO | p | p | r | n | n | r | r | r | r | r |
| SAPRTE-SEMERE | p | r | r | r | r | r | r | r | r | r |
| SAPRTE-SLOATO | p | r | r | r | r | r | r | r | r | r |
| SEMERE-BACCRA | p | r | r | r | r | r | p | p | r | r |
| SEMERE-BARRPE | r | r | r | r | n | r | r | r | r | r |
| SEMERE-CASTEC | n | n | p | p | p | p | p | n | n | p |
| SEMERE-CASTHY | n | r | p | p | r | r | r | n | r | p |
| SEMERE-CASTIN | p | p | p | r | r | r | r | p | r | p |
| SEMERE-CINNBE | p | r | p | r | r | r | r | r | r | r |
| SEMERE-DICHGE | p | r | r | r | r | r | r | r | r | n |
| SEMERE-FICULA | r | n | r | r | r | n | n | r | r | r |
| SEMERE-GARCCO | n | r | p | p | p | p | r | n | n | r |
| SEMERE-KNEMFU | p | p | r | n | n | r | r | r | r | r |
| SEMERE-LEEACO | n | r | r | r | n | r | r | r | r | r |
| SEMERE-MEZZCR | p | p | r | p | p | r | r | r | p | r |
| SEMERE-NEPHCH | p | r | r | r | r | r | r | r | r | r |
| SEMERE-PARACH | r | r | r | p | r | r | r | r | r | r |
| SEMERE-PHOELA | n | n | p | r | r | r | r | r | r | r |
| SEMERE-PITTKE | n | n | r | r | r | r | r | r | n | n |
| SEMERE-POMETO | r | r | r | r | r | r | r | r | r | r |
| SEMERE-SAPRTE | p | r | r | r | r | r | r | r | r | r |
| SEMERE-SLOATO | p | p | r | r | r | r | r | r | r | r |
| SLOATO-BACCRA | r | n | r | r | r | p | p | r | r | r |
| SLOATO-BARRPE | p | r | r | r | n | r | r | p | p | r |
| SLOATO-CASTEC | n | n | n | p | p | p | p | n | n | n |
| SLOATO-CASTHY | n | n | n | p | p | p | p | n | r | r |
| SLOATO-CASTIN | r | r | r | r | r | r | r | p | p | p |
| SLOATO-CINNBE | n | n | p | p | p | p | n | n | r | r |
| SLOATO-DICHGE | p | p | p | p | r | r | r | r | r | r |
| SLOATO-FICULA | n | n | n | r | n | p | r | r | r | r |
| SLOATO-GARCCO | n | n | n | p | p | p | p | n | n | n |
| SLOATO-KNEMFU | p | p | p | n | n | n | r | r | r | r |
| SLOATO-LEEACO | n | n | r | r | r | p | r | r | p | r |
| SLOATO-MEZZCR | p | p | p | p | n | n | n | n | r | p |
| SLOATO-NEPHCH | r | r | r | r | r | r | r | r | r | r |
| SLOATO-PARACH | p | p | p | p | r | n | n | r | r | r |
| SLOATO-PHOELA | n | n | r | r | r | r | r | r | r | r |
| SLOATO-PITTKE | n | n | n | r | p | p | p | r | n | n |
| SLOATO-POMETO | p | p | r | r | n | n | r | r | r | r |
| SLOATO-SAPRTE | p | r | r | r | r | r | r | r | r | r |
| SLOATO-SEMERE | p | p | r | r | r | r | r | r | r | r |
| **Adults** |  |  |  |  |  |  |  |  |  |  |
| BACCRA-BARRPE | p | r | r | n | n | r | r | r | r | r |
| BACCRA-CASTEC | n | n | p | p | p | r | n | n | n | n |
| BACCRA-CASTHY | n | n | n | p | p | p | p | r | n | r |
| BACCRA-CASTIN | r | p | r | n | r | r | r | r | r | r |
| BACCRA-CINNBE | p | p | r | r | r | p | r | n | r | r |
| BACCRA-DICHGE | p | r | r | r | n | n | n | p | r | r |
| BACCRAE-FICULA | p | p | r | n | n | p | p | r | n | n |
| BACCRA-GARCCO | p | r | n | n | r | n | n | r | n | n |
| BACCRA-KNEMFU | p | r | n | n | n | n | p | p | r | r |
| BACCRA-LEEACO | r | n | r | r | n | n | p | r | r | r |
| BACCRA-MEZZCR | p | p | r | p | r | r | r | r | r | p |
| BACCRA-NEPHCH | r | r | r | p | n | r | r | r | r | r |
| BACCRA-PARACH | p | p | r | r | r | r | r | p | p | r |
| BACCRA-PHOELA | r | r | r | r | r | r | n | p | r | r |
| BACCRA-PITTKE | r | r | r | n | n | p | p | n | n | r |
| BACCRA-POMETO | p | p | r | r | n | r | r | r | r | r |
| BACCRA-SAPRTE | p | r | n | r | r | n | n | p | r | r |
| BACCRA-SEMERE | p | r | r | r | r | r | r | r | r | r |
| BACCRA-SLOATO | r | r | r | r | r | r | r | r | r | r |
| BARRPE-BACCRA | p | r | r | n | n | r | p | r | p | r |
| BARRPE-CASTEC | n | n | n | p | p | p | r | n | n | n |
| BARRPE-CASTHY | n | n | n | p | p | r | r | n | r | r |
| BARRPE-CASTIN | r | r | r | n | r | r | r | r | r | r |
| BARRPE-CINNBE | n | n | p | p | p | n | r | r | r | r |
| BARRPE-DICHGE | r | p | p | p | n | n | r | r | p | p |
| BARRPE-FICULA | n | n | r | p | p | r | r | r | r | n |
| BARRPE-GARCCO | r | r | r | r | r | r | r | r | r | p |
| BARRPE-KNEMFU | r | r | r | r | n | r | r | r | r | r |
| BARRPE-LEEACO | p | n | r | n | n | r | r | r | p | p |
| BARRPE-MEZZCR | r | r | p | n | p | r | n | r | r | p |
| BARRPE-NEPHCH | n | r | r | r | n | r | n | r | p | r |
| BARRPE-PARACH | p | r | r | r | n | r | r | r | r | r |
| BARRPE-PHOELA | r | r | r | p | r | n | r | r | p | r |
| BARRPE-PITTKE | n | r | p | r | r | r | r | r | r | r |
| BARRPE-POMETO | r | r | r | r | r | r | r | r | r | r |
| BARRPE-SAPRTE | n | n | n | n | n | p | p | p | r | p |
| BARRPE-SEMERE | r | r | r | r | r | r | r | r | r | r |
| BARRPE-SLOATO | r | r | r | r | r | r | r | r | r | r |
| CASTEC-BACCRA | n | n | r | r | p | r | r | n | n | r |
| CASTEC-BARRPE | n | n | n | p | p | p | r | n | n | n |
| CASTEC-CASTHY | p | p | p | n | n | n | r | p | r | r |
| CASTEC-CASTIN | n | r | r | r | r | r | r | r | r | p |
| CASTEC-CINNBE | r | r | r | r | r | n | n | r | p | p |
| CASTEC-DICHGE | n | n | r | p | p | p | p | r | n | n |
| CASTECE-FICULA | n | n | r | r | n | n | n | r | p | p |
| CASTEC-GARCCO | r | r | p | p | r | n | n | n | r | r |
| CASTEC-KNEMFU | n | n | p | p | p | r | n | n | n | n |
| CASTEC-LEEACO | n | n | n | r | p | r | n | n | r | p |
| CASTEC-MEZZCR | n | n | n | n | r | p | p | p | r | r |
| CASTEC-NEPHCH | n | r | r | r | r | r | r | n | n | r |
| CASTEC-PARACH | n | n | n | p | p | p | p | r | n | n |
| CASTEC-PHOELA | r | r | r | r | r | r | r | r | n | n |
| CASTEC-PITTKE | r | r | p | p | n | n | n | n | r | p |
| CASTEC-POMETO | n | n | r | p | p | p | r | n | n | r |
| CASTEC-SAPRTE | n | n | n | n | n | n | r | r | r | p |
| CASTEC-SEMERE | n | r | r | r | r | r | p | r | r | r |
| CASTEC-SLOATO | n | n | n | r | r | p | p | r | n | r |
| CASTHY-BACCRA | n | n | n | p | r | p | p | r | n | n |
| CASTHY-BARRPE | n | n | r | p | p | r | r | n | n | n |
| CASTHY-CASTEC | p | p | p | r | n | n | r | p | r | r |
| CASTHY-CASTIN | n | n | r | r | r | r | r | r | p | r |
| CASTHY-CINNBE | p | r | n | n | r | r | r | p | p | p |
| CASTHY-DICHGE | n | n | n | r | r | p | p | r | r | r |
| CASTHYE-FICULA | r | r | n | n | n | r | r | p | r | r |
| CASTHY-GARCCO | p | r | r | r | r | r | n | r | r | r |
| CASTHY-KNEMFU | r | p | r | r | r | n | n | n | n | r |
| CASTHY-LEEACO | n | n | n | r | r | r | p | r | r | r |
| CASTHY-MEZZCR | n | n | n | r | r | p | p | r | n | r |
| CASTHY-NEPHCH | p | r | r | r | r | r | r | r | r | r |
| CASTHY-PARACH | n | n | n | n | n | r | r | r | r | r |
| CASTHY-PHOELA | r | p | r | r | n | r | r | r | r | r |
| CASTHY-PITTKE | p | r | r | n | n | n | n | r | r | r |
| CASTHY-POMETO | n | n | p | p | r | p | r | r | n | r |
| CASTHY-SAPRTE | n | r | r | p | r | r | r | r | r | p |
| CASTHY-SEMERE | r | r | r | r | p | r | r | r | r | r |
| CASTHY-SLOATO | r | r | r | p | p | r | r | r | r | r |
| CASTIN-BACCRA | r | r | r | n | r | r | r | r | r | r |
| CASTIN-BARRPE | r | r | r | n | r | r | r | r | r | r |
| CASTIN-CASTEC | n | r | r | r | p | p | r | r | r | p |
| CASTIN-CASTHY | n | r | r | r | p | r | r | p | r | r |
| CASTIN-CINNBE | r | r | r | n | r | r | r | r | r | r |
| CASTIN-DICHGE | p | r | r | r | r | r | r | r | r | r |
| CASTINE-FICULA | p | r | n | r | r | r | r | r | n | n |
| CASTIN-GARCCO | r | n | p | r | r | r | r | r | r | r |
| CASTIN-KNEMFU | n | r | r | r | r | r | r | r | r | n |
| CASTIN-LEEACO | r | r | r | r | r | r | n | r | r | r |
| CASTIN-MEZZCR | n | p | r | r | r | r | r | r | r | r |
| CASTIN-NEPHCH | r | n | r | r | r | r | r | r | r | r |
| CASTIN-PARACH | r | r | p | p | r | r | r | r | r | r |
| CASTIN-PHOELA | r | r | r | r | r | r | p | r | p | r |
| CASTIN-PITTKE | r | r | p | r | r | n | r | n | n | n |
| CASTIN-POMETO | p | r | r | r | r | p | r | r | r | r |
| CASTIN-SAPRTE | r | p | n | r | r | r | r | n | p | p |
| CASTIN-SEMERE | r | r | p | r | r | r | r | r | n | r |
| CASTIN-SLOATO | r | r | r | r | r | r | r | r | r | r |
| CINNBE-BACCRA | p | r | r | r | r | p | r | n | n | n |
| CINNBE-BARRPE | r | r | r | p | r | r | n | p | r | r |
| CINNBE-CASTEC | n | r | r | r | r | n | r | p | p | p |
| CINNBE-CASTHY | p | p | n | n | r | r | r | p | p | r |
| CINNBE-CASTIN | r | r | r | r | r | r | r | n | r | r |
| CINNBE-DICHGE | p | r | r | n | r | p | p | r | n | n |
| CINNBE-FICULA | p | p | n | n | r | r | r | r | r | r |
| CINNBE-GARCCO | p | p | r | n | n | r | r | p | r | r |
| CINNBE-KNEMFU | p | p | r | n | r | r | r | p | p | r |
| CINNBE-LEEACO | n | r | r | n | r | r | n | r | p | r |
| CINNBE-MEZZCR | n | n | p | p | r | r | r | r | r | r |
| CINNBE-NEPHCH | p | p | n | n | n | r | p | r | n | r |
| CINNBE-PARACH | p | n | r | r | n | n | n | n | n | n |
| CINNBE-PHOELA | r | r | r | r | r | r | r | r | p | p |
| CINNBE-PITTKE | p | p | r | n | n | r | p | p | r | r |
| CINNBE-POMETO | n | n | r | p | p | p | r | n | n | n |
| CINNBE-SAPRTE | p | r | r | r | r | r | r | r | r | n |
| CINNBE-SEMERE | r | r | r | r | r | r | r | r | r | r |
| CINNBE-SLOATO | n | r | p | p | r | r | n | n | n | n |
| DICHGE-BACCRA | p | p | r | p | n | n | r | p | n | r |
| DICHGE-BARRPE | r | r | p | p | n | n | n | r | p | p |
| DICHGE-CASTEC | n | n | r | p | p | r | r | r | r | n |
| DICHGE-CASTHY | n | n | r | p | r | p | r | r | r | r |
| DICHGE-CASTIN | p | r | r | r | r | r | r | r | r | r |
| DICHGE-CINNBE | p | r | r | r | r | r | p | r | n | n |
| DICHGE-FICULA | r | r | r | p | p | r | r | n | n | n |
| DICHGE-GARCCO | r | p | r | r | r | r | n | r | r | r |
| DICHGE-KNEMFU | p | r | r | r | n | n | r | p | p | p |
| DICHGE-LEEACO | r | r | r | r | r | r | r | r | r | n |
| DICHGE-MEZZCR | r | p | p | r | r | n | n | r | p | r |
| DICHGE-NEPHCH | p | p | r | n | r | r | r | r | r | r |
| DICHGE-PARACH | p | p | p | r | r | n | n | r | p | p |
| DICHGE-PHOELA | r | r | r | r | n | p | r | r | r | r |
| DICHGE-PITTKE | p | p | n | r | r | r | p | r | r | r |
| DICHGE-POMETO | p | p | p | r | n | r | r | r | p | r |
| DICHGE-SAPRTE | r | p | r | n | r | p | r | p | r | r |
| DICHGE-SEMERE | p | p | r | r | n | n | p | r | r | p |
| DICHGE-SLOATO | r | p | p | r | r | n | n | n | r | r |
| FICULA-BACCRA | r | p | p | t | n | p | p | r | n | n |
| FICULA-BARRPE | r | r | r | p | p | n | r | r | r | n |
| FICULA-CASTEC | n | n | p | p | r | n | n | r | p | p |
| FICULA-CASTHY | r | r | n | n | r | r | p | p | r | r |
| FICULA-CASTIN | p | r | n | r | r | r | r | r | n | r |
| FICULA-CINNBE | p | p | r | n | r | r | r | r | r | r |
| FICULA-DICHGE | r | r | n | r | p | p | p | n | n | n |
| FICULA-GARCCO | p | p | r | n | r | r | p | p | r | r |
| FICULA-KNEMFU | r | r | n | n | n | r | r | r | p | r |
| FICULA-LEEACO | n | r | r | r | r | r | n | n | n | p |
| FICULA-MEZZCR | n | p | p | p | r | n | n | n | r | r |
| FICULA-NEPHCH | p | r | n | n | r | r | r | r | r | r |
| FICULA-PARACH | r | p | r | r | p | n | n | n | n | n |
| FICULA-PHOELA | r | r | r | r | r | r | r | r | r | p |
| FICULA-PITTKE | p | p | p | n | n | r | p | r | r | n |
| FICULA-POMETO | n | r | r | p | p | p | n | n | n | n |
| FICULA-SAPRTE | n | n | r | r | r | p | r | n | r | r |
| FICULA-SEMERE | p | r | r | r | r | r | r | r | r | r |
| FICULA-SLOATO | n | r | p | p | p | r | n | n | r | r |
| GARCCO-BACCRA | p | r | r | r | r | p | p | r | r | r |
| GARCCO-BARRPE | r | r | r | r | r | r | r | p | p | p |
| GARCCO-CASTEC | r | r | p | p | p | r | n | n | r | r |
| GARCCO-CASTHY | p | n | r | r | r | r | r | r | r | r |
| GARCCO-CASTIN | n | n | r | r | r | r | r | r | r | r |
| GARCCO-CINNBE | p | r | n | n | n | r | r | p | p | r |
| GARCCO-DICHGE | r | p | r | n | r | r | n | n | r | r |
| GARCCO-FICULA | p | r | r | n | r | r | p | p | p | r |
| GARCCO-KNEMFU | p | p | r | r | n | n | r | p | r | r |
| GARCCO-LEEACO | r | p | r | n | n | r | r | r | p | r |
| GARCCO-MEZZCR | r | n | p | r | r | r | r | r | r | r |
| GARCCO-NEPHCH | r | p | r | r | r | r | r | r | r | r |
| GARCCO-PARACH | r | p | r | r | r | r | p | p | r | n |
| GARCCO-PHOELA | r | r | r | r | r | r | r | p | r | r |
| GARCCO-PITTKE | p | p | n | n | r | r | r | r | r | r |
| GARCCO-POMETO | r | r | r | p | r | p | r | n | n | n |
| GARCCO-SAPRTE | p | p | r | n | n | r | r | r | n | n |
| GARCCO-SEMERE | r | r | p | r | r | r | r | r | p | r |
| GARCCO-SLOATO | r | r | r | r | r | r | r | r | r | n |
| PITTKE-CASTIN | r | r | p | r | r | n | r | r | r | r |
| KNEMFU-BACCRA | p | r | n | n | n | n | p | p | r | r |
| KNEMFU-BARRPE | r | r | r | n | n | r | r | r | r | r |
| KNEMFU-CASTEC | n | n | p | p | p | r | n | n | n | n |
| KNEMFU-CASTHY | r | p | r | r | r | n | r | n | n | r |
| KNEMFU-CASTIN | n | p | p | r | r | r | r | r | r | n |
| KNEMFU-CINNBE | p | r | r | n | r | r | r | p | p | p |
| KNEMFU-DICHGE | p | r | r | r | n | n | r | p | p | p |
| KNEMFU-FICULA | r | r | n | n | n | p | p | p | p | r |
| KNEMFU-GARCCO | p | p | r | r | n | r | r | p | r | r |
| KNEMFU-LEEACO | n | n | r | r | n | n | r | n | p | p |
| KNEMFU-MEZZCR | r | r | r | r | r | r | r | p | p | r |
| KNEMFU-NEPHCH | p | r | r | n | r | r | r | r | r | p |
| KNEMFU-PARACH | r | p | r | r | n | n | r | p | p | r |
| KNEMFU-PHOELA | p | p | r | n | r | r | r | r | r | r |
| KNEMFU-PITTKE | p | r | r | r | r | n | n | r | r | r |
| KNEMFU-POMETO | r | p | r | r | r | r | p | r | r | r |
| KNEMFU-SAPRTE | p | r | r | n | n | r | p | p | p | n |
| KNEMFU-SEMERE | r | r | r | r | n | r | r | p | r | p |
| KNEMFU-SLOATO | r | r | r | r | r | n | r | r | r | r |
| LEEACO-BACCRA | r | n | r | r | n | n | p | r | r | r |
| LEEACO-BARRPE | p | n | r | n | r | r | r | n | p | p |
| LEEACO-CASTEC | n | n | n | n | r | r | n | n | r | r |
| LEEACO-CASTHY | n | n | n | r | r | r | r | r | n | r |
| LEEACO-CASTIN | r | r | r | r | n | r | n | r | r | r |
| LEEACO-CINNBE | r | r | r | r | r | r | r | r | p | p |
| LEEACO-DICHGE | r | r | r | r | r | r | r | r | r | r |
| LEEACO-FICULA | n | r | r | r | r | r | n | n | r | p |
| LEEACO-GARCCO | r | r | r | n | n | r | r | r | r | r |
| LEEACO-KNEMFU | r | r | r | r | n | n | r | r | r | r |
| LEEACO-MEZZCR | r | r | r | n | n | r | p | p | n | r |
| LEEACO-NEPHCH | r | r | r | n | n | r | r | r | r | p |
| LEEACO-PARACH | r | n | r | n | n | r | r | r | p | r |
| LEEACO-PHOELA | r | r | r | r | r | r | r | r | p | p |
| LEEACO-PITTKE | r | r | p | r | r | n | n | r | r | r |
| LEEACO-POMETO | n | r | n | r | n | r | r | r | r | r |
| LEEACO-SAPRTE | r | n | n | r | r | r | r | r | r | r |
| LEEACO-SEMERE | r | r | r | r | r | n | r | r | r | n |
| LEEACO-SLOATO | r | r | r | r | n | r | r | p | p | p |
| MEZZCR-BACCRA | p | p | r | r | r | r | r | r | n | r |
| MEZZCR-BARRPE | r | r | p | n | p | r | n | p | r | p |
| MEZZCR-CASTEC | n | n | n | n | p | p | p | r | n | n |
| MEZZCR-CASTHY | n | n | n | r | r | p | p | r | n | n |
| MEZZCR-CASTIN | n | p | r | r | r | r | r | r | r | r |
| MEZZCR-CINNBE | n | n | p | p | r | r | r | r | r | r |
| MEZZCR-DICHGE | r | p | p | r | r | n | n | n | p | r |
| MEZZCRE-FICULA | n | p | p | p | n | n | n | r | p | p |
| MEZZCR-GARCCO | r | n | p | r | r | r | r | r | r | r |
| MEZZCR-KNEMFU | n | n | r | p | r | n | n | p | p | r |
| MEZZCR-LEEACO | p | p | r | n | r | r | r | r | n | n |
| MEZZCR-NEPHCH | n | r | r | r | r | n | r | r | r | r |
| MEZZCR-PARACH | r | p | p | r | n | n | n | r | r | p |
| MEZZCR-PHOELA | n | n | r | r | r | r | p | r | p | r |
| MEZZCR-PITTKE | r | n | n | p | r | n | n | r | p | n |
| MEZZCR-POMETO | p | p | r | r | r | r | r | n | r | r |
| MEZZCR-SAPRTE | r | r | r | p | n | n | r | r | r | r |
| MEZZCR-SEMERE | p | r | r | r | r | r | r | r | r | p |
| MEZZCR-SLOATO | p | p | r | r | r | n | n | r | r | r |
| NEPHCH-BACCRA | n | p | r | p | r | r | r | r | r | r |
| NEPHCH-BARRPE | n | r | p | r | n | r | r | p | p | r |
| NEPHCH-CASTEC | n | n | n | p | p | p | p | n | n | n |
| NEPHCH-CASTHY | p | r | r | r | r | p | r | r | r | r |
| NEPHCH-CASTIN | r | n | r | p | r | r | r | r | r | n |
| NEPHCH-CINNBE | p | p | n | n | n | r | p | r | r | r |
| NEPHCH-DICHGE | p | p | p | r | n | r | p | r | p | r |
| NEPHCH-FICULA | p | r | r | n | r | r | p | r | r | r |
| NEPHCH-GARCCO | r | p | r | r | r | r | r | r | p | p |
| NEPHCH-KNEMFU | p | r | r | n | n | n | r | r | r | r |
| NEPHCH-LEEACO | r | r | r | r | n | r | r | r | r | p |
| NEPHCH-MEZZCR | n | r | r | p | r | n | n | p | p | r |
| NEPHCH-PARACH | r | p | r | r | r | r | r | r | r | r |
| NEPHCH-PHOELA | r | r | r | r | r | r | p | r | r | p |
| NEPHCH-PITTKE | p | r | r | n | n | r | r | r | r | r |
| NEPHCH-POMETO | r | r | p | r | r | r | n | r | p | r |
| NEPHCH-SAPRTE | r | r | r | r | p | r | n | n | n | r |
| NEPHCH-SEMERE | p | r | r | r | p | r | r | r | r | r |
| NEPHCH-SLOATO | r | r | r | r | r | n | r | r | r | r |
| PARACH-BACCRA | p | r | r | r | r | r | r | r | p | r |
| PARACH-BARRPE | p | r | r | r | n | r | r | r | r | r |
| PARACH-CASTEC | n | n | n | n | r | r | r | n | r | r |
| PARACH-CASTHY | n | n | n | p | p | p | p | p | r | r |
| PARACH-CASTIN | n | n | p | p | r | r | r | r | r | r |
| PARACH-CINNBE | n | n | n | n | n | n | n | n | n | n |
| PARACH-DICHGE | p | p | p | r | r | n | n | r | p | p |
| PARACH-FICULA | n | n | n | n | n | n | n | n | n | p |
| PARACH-GARCCO | r | p | r | r | r | r | r | r | r | n |
| PARACH-KNEMFU | r | p | r | r | n | n | r | p | p | r |
| PARACH-LEEACO | r | n | r | n | r | r | r | r | p | r |
| PARACH-MEZZCR | p | p | p | r | r | n | n | r | r | p |
| PARACH-NEPHCH | r | r | r | r | r | r | r | r | r | r |
| PARACH-PHOELA | r | r | r | r | p | r | p | r | r | r |
| PARACH-PITTKE | r | r | r | r | r | r | r | r | r | n |
| PARACH-POMETO | p | p | r | r | r | r | r | r | r | r |
| PARACH-SAPRTE | r | p | r | r | r | r | r | p | r | r |
| PARACH-SEMERE | r | r | r | r | r | r | r | r | r | r |
| PARACH-SLOATO | p | p | r | r | r | r | r | r | r | r |
| PHOELA-BACCRA | n | r | r | r | r | r | n | p | r | r |
| PHOELA-BARRPE | r | r | r | p | n | n | r | r | p | r |
| PHOELA-CASTEC | r | r | p | p | p | p | r | n | n | n |
| PHOELA-CASTHY | r | p | p | r | n | r | r | r | n | r |
| PHOELA-CASTIN | r | r | p | r | n | r | r | r | p | r |
| PHOELA-CINNBE | r | r | r | n | r | r | n | r | p | p |
| PHOELA-DICHGE | n | n | p | r | n | p | r | r | r | n |
| PHOELA-FICULA | r | r | r | n | r | r | r | r | r | r |
| PHOELA-GARCCO | r | p | p | p | r | n | n | r | p | r |
| PHOELA-KNEMFU | p | p | r | n | r | r | n | n | r | r |
| PHOELA-LEEACO | r | p | r | n | r | r | r | r | p | p |
| PHOELA-MEZZCR | n | n | p | r | r | r | p | r | p | r |
| PHOELA-NEPHCH | r | r | n | r | r | r | r | r | r | r |
| PHOELA-PARACH | r | r | r | r | p | r | p | r | r | r |
| PHOELA-PITTKE | r | p | r | n | r | n | r | n | n | p |
| PHOELA-POMETO | n | n | r | r | r | r | r | r | r | r |
| PHOELA-SAPRTE | r | r | r | p | p | n | n | p | r | r |
| PHOELA-SEMERE | r | r | r | r | r | p | n | n | r | r |
| PHOELA-SLOATO | n | p | r | r | p | r | r | r | r | p |
| PITTKE-BACCRA | r | r | r | n | n | p | p | n | r | r |
| PITTKE-BARRPE | n | r | p | r | r | r | r | r | r | r |
| PITTKE-CASTEC | n | r | p | p | r | n | n | n | p | p |
| PITTKE-CASTHY | p | p | r | n | n | r | r | r | p | r |
| PITTKE-CINNBE | p | p | n | n | n | r | p | p | r | r |
| PITTKE-DICHGE | p | r | n | r | r | r | r | r | r | r |
| PITTKEE-FICULA | p | p | r | n | n | r | p | r | r | n |
| PITTKE-GARCCO | p | p | r | n | n | r | r | r | r | n |
| PITTKE-KNEMFU | p | r | r | r | r | r | n | n | n | n |
| PITTKE-LEEACO | r | r | p | p | p | r | n | r | n | r |
| PITTKE-MEZZCR | n | n | p | p | r | n | r | r | p | n |
| PITTKE-NEPHCH | p | r | r | n | n | r | r | r | r | r |
| PITTKE-PARACH | r | r | r | r | p | r | r | p | r | n |
| PITTKE-PHOELA | r | p | r | r | r | r | r | r | r | p |
| PITTKE-POMETO | r | r | r | p | r | r | r | p | r | n |
| PITTKE-SAPRTE | r | r | p | r | n | r | r | r | r | r |
| PITTKE-SEMERE | p | r | r | r | r | n | r | r | r | r |
| PITTKE-SLOATO | r | n | r | p | p | r | r | r | r | n |
| POMETO-BACCRA | p | p | r | n | r | r | r | r | n | r |
| POMETO-BARRPE | r | r | r | r | r | r | r | r | r | r |
| POMETO-CASTEC | n | n | r | p | p | p | r | n | n | r |
| POMETO-CASTHY | n | n | p | p | r | p | r | r | n | r |
| POMETO-CASTIN | r | r | r | r | r | r | r | r | r | r |
| POMETO-CINNBE | r | r | r | p | p | p | r | n | n | n |
| POMETO-DICHGE | p | p | p | r | r | n | r | r | r | r |
| POMETO-FICULA | n | n | r | p | r | p | r | r | n | n |
| POMETO-GARCCO | r | r | r | p | r | r | r | r | n | n |
| POMETO-KNEMFU | r | p | r | r | r | n | r | r | r | r |
| POMETO-LEEACO | n | r | r | p | r | r | r | r | r | r |
| POMETO-MEZZCR | p | p | r | r | r | r | r | n | r | p |
| POMETO-NEPHCH | n | r | p | r | r | r | n | r | r | r |
| POMETO-PARACH | p | p | p | r | r | r | r | r | r | r |
| POMETO-PHOELA | r | n | r | p | r | r | r | r | r | r |
| POMETO-PITTKE | r | r | r | p | r | r | r | r | r | n |
| POMETO-SAPRTE | p | p | n | r | r | r | r | r | r | r |
| POMETO-SEMERE | r | r | r | r | r | r | r | r | r | r |
| POMETO-SLOATO | p | p | r | r | r | r | r | r | r | r |
| SAPRTE-BACCRA | p | p | n | r | r | n | r | p | r | r |
| SAPRTE-BARRPE | n | n | n | n | n | p | p | p | r | p |
| SAPRTE-CASTEC | n | n | n | r | r | r | r | r | r | r |
| SAPRTE-CASTHY | n | r | r | p | r | r | r | r | r | p |
| SAPRTE-CASTIN | r | r | r | p | r | r | r | r | r | p |
| SAPRTE-CINNBE | p | r | r | r | r | r | r | r | r | n |
| SAPRTE-DICHGE | r | r | p | n | r | p | r | r | r | n |
| SAPRTE-FICULA | r | r | p | r | r | p | r | r | r | r |
| SAPRTE-GARCCO | p | p | r | r | r | r | p | r | n | n |
| SAPRTE-KNEMFU | p | r | r | n | n | r | r | r | r | r |
| SAPRTE-LEEACO | r | n | r | r | r | p | r | r | r | r |
| SAPRTE-MEZZCR | r | r | r | p | n | r | r | r | r | r |
| SAPRTE-NEPHCH | r | r | r | p | p | r | n | n | n | r |
| SAPRTE-PARACH | r | r | r | r | r | r | r | r | r | r |
| SAPRTE-PHOELA | n | r | r | p | p | r | n | p | r | r |
| SAPRTE-PITTKE | r | r | p | r | n | p | r | r | r | r |
| SAPRTE-POMETO | p | p | n | r | p | r | r | r | r | r |
| SAPRTE-SEMERE | r | r | r | r | p | r | r | r | r | r |
| SAPRTE-SLOATO | r | r | r | r | r | p | r | p | p | r |
| SEMERE- POMETO | r | r | r | r | r | r | r | r | r | r |
| SEMERE-BACCRA | p | r | r | r | r | r | r | r | r | r |
| SEMERE-BARRPE | r | r | r | r | r | r | r | r | r | r |
| SEMERE-CASTEC | n | n | p | r | r | p | p | r | r | r |
| SEMERE-CASTHY | r | r | r | r | p | r | r | p | r | r |
| SEMERE-CASTIN | r | p | r | r | r | r | r | r | r | r |
| SEMERE-CINNBE | r | r | r | r | r | r | r | r | r | r |
| SEMERE-DICHGE | p | p | r | r | n | n | r | r | r | p |
| SEMERE-FICULA | p | r | r | n | r | r | r | r | r | r |
| SEMERE-GARCCO | n | r | p | r | r | r | r | r | n | r |
| SEMERE-KNEMFU | p | r | r | r | n | r | r | p | r | p |
| SEMERE-LEEACO | n | r | r | r | r | n | r | n | n | n |
| SEMERE-MEZZCR | p | r | r | r | r | r | r | r | r | r |
| SEMERE-NEPHCH | p | r | r | r | p | r | n | r | p | r |
| SEMERE-PARACH | r | r | r | r | r | r | r | p | r | r |
| SEMERE-PHOELA | r | r | r | r | r | p | r | r | r | p |
| SEMERE-PITTKE | p | r | r | r | r | n | r | r | r | r |
| SEMERE-SAPRTE | r | p | p | r | n | r | r | r | r | r |
| SEMERE-SLOATO | r | r | r | r | r | r | r | r | r | r |
| SLOATO-BACCRA | r | n | r | r | r | p | p | r | r | r |
| SLOATO-BARRPE | r | r | r | r | r | r | r | r | r | r |
| SLOATO-CASTEC | n | n | n | r | r | p | p | r | n | r |
| SLOATO-CASTHY | r | r | r | r | p | r | r | r | r | r |
| SLOATO-CASTIN | r | r | r | r | r | r | r | r | r | p |
| SLOATO-CINNBE | n | r | r | p | r | r | r | r | r | r |
| SLOATO-DICHGE | r | p | p | r | r | r | n | r | r | r |
| SLOATO-FICULA | n | r | p | p | p | r | n | r | r | r |
| SLOATO-GARCCO | r | r | r | r | r | rr | r | r | r | n |
| SLOATO-KNEMFU | n | r | r | r | r | n | r | r | r | r |
| SLOATO-LEEACO | n | r | r | r | n | r | r | p | r | r |
| SLOATO-MEZZCR | p | p | r | r | r | r | n | r | r | r |
| SLOATO-NEPHCH | r | r | r | r | p | n | r | r | r | r |
| SLOATO-PARACH | p | p | r | r | r | r | r | r | r | r |
| SLOATO-PHOELA | r | r | r | r | r | r | r | r | r | p |
| SLOATO-PITTKE | r | n | r | p | p | r | r | r | r | n |
| SLOATO-POMETO | p | p | r | r | r | r | r | r | r | r |
| SLOATO-SAPRTE | r | r | r | r | r | r | r | r | r | r |
| SLOATO-SEMERE | r | r | r | r | r | r | r | r | r | r |
